# Syntaxin 3B Mediates Light‐Dependent Interactions with STXBP1 and Arrestin 4: Distinct Roles in Rods and Cones

**DOI:** 10.1002/advs.202513319

**Published:** 2025-11-12

**Authors:** Lars Tebbe, Larissa Ikelle, Mustafa S. Makia, Mashal Kakakhel, Muayyad R. Al‐Ubaidi, Muna I. Naash

**Affiliations:** ^1^ Department of Biomedical Engineering University of Houston Houston TX 77204 USA

**Keywords:** cone arrestin, retina, syntaxin, syntaxin 3B, syntaxin binding protein 1

## Abstract

Syntaxin 3 (STX3), a member of the soluble N‐ethylmaleimide‐sensitive factor attachment protein receptor (SNARE) family, plays a central role in vesicle fusion. Beyond its synaptic localization, STX3 is also detected in the photoreceptor inner segment, where its function remains poorly understood. It is shown that STX3 interacts with rhodopsin, peripherin 2, and the rod outer segment protein 1. In rod‐specific STX3 knockout retinas, these proteins are mislocalized, whereas cone opsins remain properly localized, suggesting a distinct STX3 function in cones. To further define its cone‐specific dysfunction, a cone‐specific STX3 knockout mouse is generated. This model exhibited early cone dysfunction followed by progressive rod impairment and photoreceptor degeneration. Cone degeneration correlated with early abnormalities in the connecting cilium. Specifically, a selective depletion of syntaxin binding protein 1 (STXBP1) and cone arrestin 4 is observed, a phenotype not seen when STX3 is specifically eliminated in rods. A light‐dependent complex comprising STX3, STXBP1, and arrestin 4 is further identified, with arrestin 4 preferentially associating with STX3 in the dark‐adapted retina and with STXBP1 in the light‐adapted retina. These findings reveal a cone‐specific, light‐regulated protein interaction network essential for cone function and survival, highlighting distinct and context‐dependent roles of STX3 in rods and cones.

## Graphical abstract

The present study investigates a cone‐specific STX3 knockout model, displaying a complete loss of cone function, a later onset reduction in rod function, and photoreceptor cell death. A cone‐specific depletion of STXBP1 and ARR4 could be observed in this model. STXBP1 preferentially bound to STX3 in light‐adapted and ARR4 in the dark‐adapted retina.

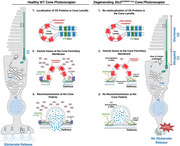

## 1 Introduction

Syntaxins, comprising 17 distinct members identified so far,^[^ ^1^ ^]^ are part of the soluble N‐ethylmaleimide‐sensitive factor attachment protein (SNAP) receptor (SNARE) superfamily.^[^ ^1^, ^2^ ^]^ SNARE proteins play a crucial role in mediating vesicle fusion with target membranes, which is essential for numerous cellular processes. This includes protein transport from the endoplasmic reticulum to the Golgi apparatus,^[^ ^3^, ^4^ ^]^ autophagy,^[^ ^5^, ^6^ ^]^ neurotransmitter release at the neuronal synapses,^[^ ^7^, ^8^, ^9^, ^10^, ^11^ ^]^ and at the specialized ribbon synapses found in photoreceptors and hair cells.^[^ ^12^, ^13^, ^14^ ^]^ Syntaxins are particularly important in regulating the formation of the SNARE complex, which subsequently triggers membrane fusion.^[^ ^15^ ^]^ The N‐terminal H_abc_‐motif of syntaxins mediates complex assembly and enables the proteins to adapt both closed (inactive) and open (active) conformations, carefully controlling the fusogenic process.^[^ ^16^, ^17^ ^]^

Syntaxin 3 (STX3) and Syntaxin 4 (STX4) are the only syntaxins expressed in the outer plexiform layer (OPL) of the retina.^[^ ^18^, ^19^, ^20^, ^21^ ^]^ Of the four known isoforms of STX3 (A, B, C, and D), only isoform B is expressed in the OPL.^[^ ^22^ ^]^ At the OPL, STX3B is essential for facilitating vesicle release at ribbon synapses, specialized synapses found exclusively in sensory cells, such as photoreceptors and hair cells.^[^ ^23^ ^]^ The SNARE complex mediating vesicle release at the ribbon synapses in photoreceptor cells consists of STX3B, VAMP2, and SNAP25.^[^ ^18^, ^19^, ^20^ ^]^ Interestingly, STX3B is also expressed in the inner segment (IS) of retinal photoreceptor cells.^[^ ^19^ ^]^ While the role of STX3B in the OPL ribbon synapses is well studied, its function in the IS remains less understood. Studies conducted in *Rana berlandieri* identified a SNARE complex consisting of STX3B, VAMP7, and SNAP25 to be involved in the transport of rhodopsin (RHO) to the photoreceptor outer segment (OS).^[^ ^24^, ^25^ ^]^ Additionally, our previous studies demonstrated an interaction between STX3 and RHO, and partial mislocalization of RHO to the IS in the photoreceptor‐specific STX3 knockout (*Stx3^f/f(CRX‐Cre)^*), further supporting STX3's role in RHO transport to the OS.^[^ ^26^, ^27^ ^]^

In addition to RHO, we previously identified peripherin 2 (PRPH2, also known as RDS) as another OS protein that interacts with STX3.^[^ ^26^ ^]^ PRPH2 is a photoreceptor‐specific tetraspanin protein localized exclusively to the disc rim membranes in the photoreceptor OS and is essential for both the formation and structural maintenance of the OS.^[^ ^28^, ^29^, ^30^, ^31^, ^32^, ^33^, ^34^ ^]^ The interaction site between PRPH2 and STX3B is localized at the C‐terminus of PRPH2 and at the SNARE domain of STX3B.^[^ ^27^ ^]^ In the *Stx3^f/f(CRX‐Cre)^* retina, both PRPH2 and its interactor, rod outer segment membrane protein 1 (ROM1), as well as RHO, were mislocalized to the IS.^[^ ^27^ ^]^ However, cone opsins were properly localized to the cone OS in the retinas of *Stx3^f/f(CRX‐Cre)^* mice, suggesting that the transport mechanisms for the photopigments differ between rods and cones.^[^ ^27^ ^]^ We also identified a novel protein complex consisting of PRPH2, STX3B, SNAP25, and syntaxin‐binding protein 1 (STXBP1).^[^ ^27^ ^]^ STXBP1, a known interactor of STX3, regulates the transition between the open and closed conformation of syntaxins and mediates vesicle fusion events.^[^ ^35^, ^36^, ^37^, ^38^, ^39^, ^40^, ^41^, ^42^ ^]^ This complex was found to localize to both the IS and OPL in wild‐type (WT) mouse rods.^[^ ^27^, ^43^ ^]^

In this study, we investigated the impact of STX3 depletion specifically in cones using Cre‐recombinase driven by the cone‐specific human red/green opsin promoter (HRGP) (*Stx3^f/f(HRGP‐Cre)^*).^[^ ^44^ ^]^ The loss of STX3 in cones led to a complete absence of cone responses, early‐onset cone photoreceptor degeneration, and structural abnormalities in the connecting cilium (CC). Furthermore, cone‐specific loss of STX3 led to the depletion of cone arrestin 4 (ARR4). Despite the selective removal of STX3 from cones, rod photoreceptors were also affected, as evidenced by a significant reduction in rod responses and widespread photoreceptor loss‐exceeding what would be expected from the mere absence of cones, which only constitute ≈3% of all photoreceptors in the mouse retina.^[^ ^45^ ^]^ These findings highlight the distinct roles of STX3 in rods and cones, demonstrating that STX3 depletion in cones and the resulting cone degeneration negatively affect rod photoreceptor health and function.

## 2 Results

### 2.1 HRGP‐Cre Induces Cone‐Specific Knockout of STX3

**Figure 1:**
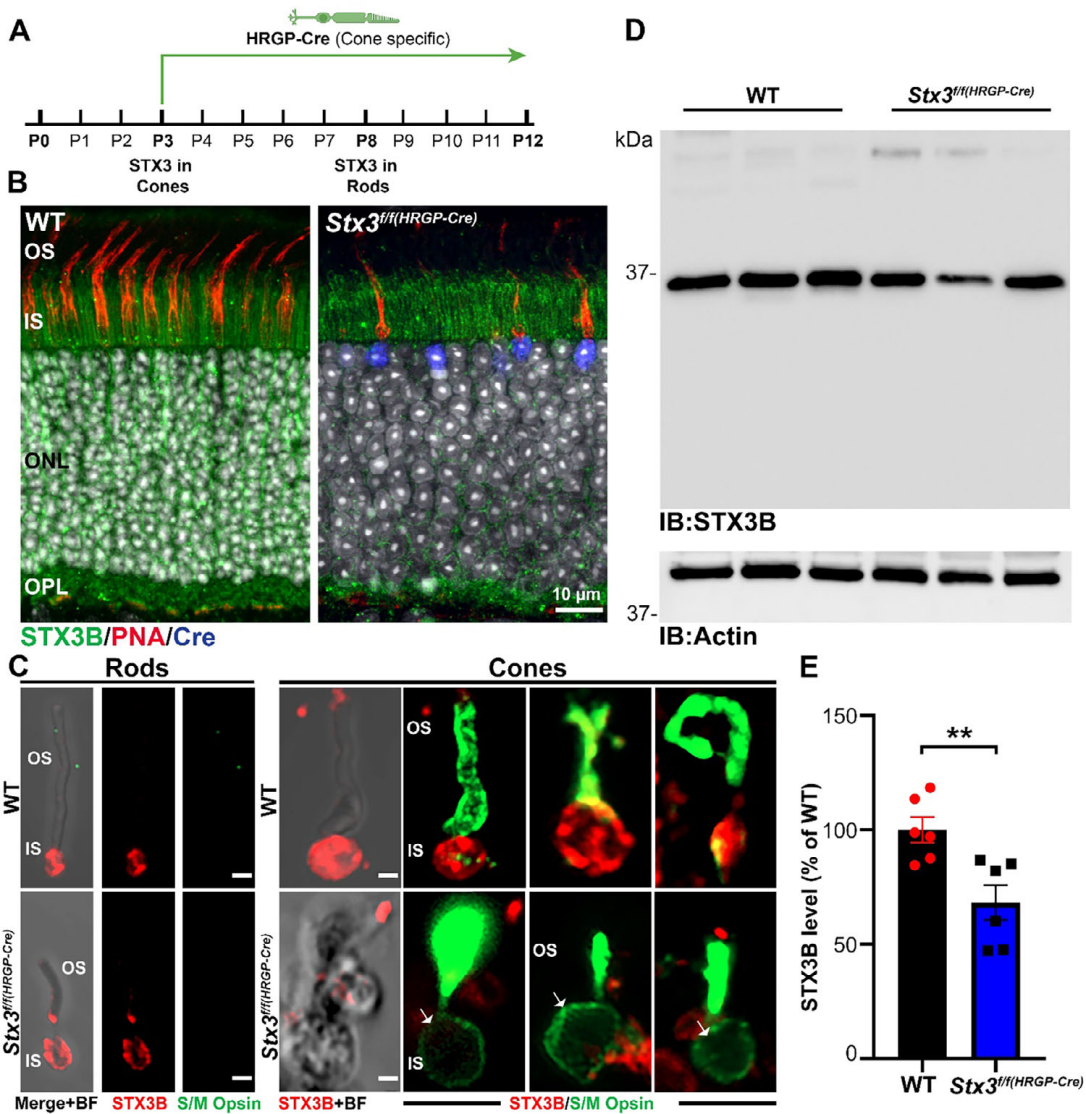
STX3B is absent in cones of the *Stx3^f/f(HRGP‐Cre)^* model. A) Diagram showing the timeline of HRGP‐Cre expression in the *Stx3^f/f(HRGP‐Cre)^* model. B) Immunofluorescence images of WT and *Stx3^f/f(HRGP‐Cre)^* retinas. In WT retinas, STX3B localizes to rod and cone cell bodies in the IS and to the photoreceptor synapses in the OPL (left). In the *Stx3^f/f(HRGP‐Cre)^* retina, STX3B is missing from cone cell bodies and synapses (right). Cre expression in cones of the *Stx3^f/f(HRGP‐Cre)^* retina is also shown. C) Immunofluorescence images of isolated rods and cones from WT and *Stx3^f/f(HRGP‐Cre)^* retinas. In WT, STX3B localizes to the cell bodies of both rods and cones, whereas in *Stx3^f/f(HRGP‐Cre)^* retina, STX3B is specifically absent from cone cell bodies but remains in rod cell bodies. Scale bar is 0.5 µm. D) Representative immunoblot (IB) of P30 WT and *Stx3^f/f(HRGP‐Cre)^* retinas probed for STX3B and actin. Six independent retinas per genotype were analyzed. E) Quantification of STX3B levels in the P30 WT and *Stx3^f/f(HRGP‐Cre)^* retinas, normalized to actin and shown as a percentage of WT ± SEM. P‐value: 0.0072. Abbreviations: IB: Immunoblot; OS: outer segment; IS: inner segment; ONL: outer nuclear layer; OPL: outer plexiform layer. The scheme of the cone photoreceptor in Figure 1A was created with BioRender.com.

Given the lethality of a global *Stx3* knockout,^[^ ^46^ ^]^ we used the cone‐specific *HRGP‐Cre* to conditionally knock out STX3 in cones to assess its specific role.^[^ ^44^ ^]^ The LoxP sites were inserted flanking exons 4 and 5,^[^ ^27^ ^]^ which are present in all four STX3 isoforms (A‐D),^[^ ^20^ ^]^ enabling the complete knockout of all isoforms. As this knockout affects all isoforms, henceforth, we describe STX3 unless an antibody specific to STX3B is used. However, evidence describes STX3B as the only isoform expressed in photoreceptors.^[^ ^22^ ^]^ *HRGP‐Cre* activity in cones begins at postnatal day 3 (P3) (**Figure** **1**A).^[^ ^44^ ^]^ Paraffin sections of retinas from *HRGP‐Cre* (*Stx3^f/f(HRGP‐Cre)^*) mice were co‐labeled for STX3 (green), Cre (blue), and peanut agglutinin (PNA, red), confirming the cone‐specific *Cre* expression and depletion of STX3 in *Stx3^f/f(HRGP‐Cre)^* cones (Figure 1B). Isolation of cones from both WT and *Stx3^f/f(HRGP‐Cre)^* retinas at P30 confirmed the selective absence of STX3 in the cell body of cones in the *Stx3^f/f(HRGP‐Cre)^* retina, while rods retained normal STX3 (Figure 1C). Immunoblot (IB) analysis of P30 WT and *Stx3^f/f(HRGP‐Cre)^* retinas showed a 31.8% reduction in STX3 levels in *Stx3^f/f(HRGP‐Cre)^* retinas compared to WT (Figure 1D,E).

### 2.2 Stx3^f/f(HRGP‐Cre)^ Mice Exhibit Reduced Photoreceptor Response and Progressive Degeneration

**Figure 2:**
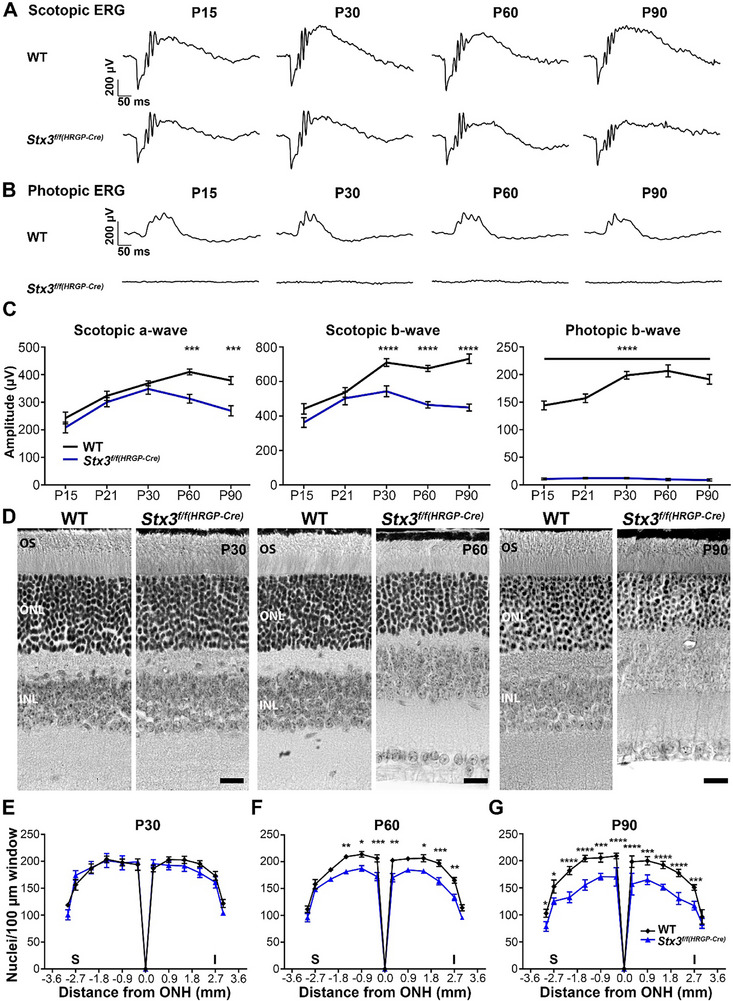
Depletion of STX3 in cones leads to cone loss and a late‐onset decline in rod function. A) Representative scotopic ERG traces from WT and *Stx3^f/f(HRGP‐Cre)^* mice at P15, P30, P60, and P90. B) Representative photopic ERG traces from WT and *Stx3^f/f(HRGP‐Cre)^* mice at P15, P30, P60, and P90. C) Maximum amplitudes of scotopic a‐ and b‐waves, and photopic b‐waves in WT and *Stx3^f/f(HRGP‐Cre)^* mice at the indicated ages. Photopic b‐wave was absent in the *Stx3^f/f(HRGP‐Cre)^* mice at all timepoints investigated. Significant reduction in scotopic a‐ and b‐waves was detected beginning at P60 and P90, respectively. N‐values: Scotopic a‐wave WT: P15: 18; P21: 24; P30: 18; P60: 20; P 90: 18. Scotopic a‐wave *Stx3^f/f(HRGP‐Cre)^*: P15: 8; P21: 10; P30: 20; P60: 18; P 90: 13. Scotopic b‐wave WT: P15: 20; P21: 28; P30: 20; P60: 20; P 90: 18. Scotopic b‐wave *Stx3^f/f(HRGP‐Cre)^*: P15: 8; P21: 12; P30: 20; P60: 23; P 90: 16. Photopic b‐wave WT: P15: 20; P21: 28; P30: 22; P60: 20; P 90: 16. Photopic b‐wave *Stx3^f/f(HRGP‐Cre)^*: P15: 8; P21: 12; P30: 20; P60: 10; P 90: 14. Data presented as mean ± SEM. P‐values for significant findings: Scotopic a‐wave: P60: 0.0001; P90: 0.0001. Scotopic b‐wave: P30: <0.0001; P60: <0.0001; P90: <0.0001. Photopic b‐wave: for all investigated timepoints: <0.0001. P‐values determined by two‐way ANOVA with Sidak's post hoc comparison. D) Representative retinal histology images from WT and *Stx3^f/f(HRGP‐Cre)^* mice at P30, P60, and P90 (scale bar: 20 µm) demonstrate the thinning of the ONL in the *Stx3^f/f(HRGP‐Cre)^* model, starting at P60. E‐G) Quantification of ONL nuclei at P30 (E), P60 (F), and P90 (G) showing significant photoreceptor loss beginning at P60 and progressing by P90. Nuclei were counted within an area of 100 x 100 µm within the ONL. N‐values: WT: 4 mice for P30 and P60, 3 mice for P90. *Stx3^f/f(HRGP‐Cre)^*: 3 mice for P30, P60, and P90. Data presented as mean ± SEM. P‐values for significant findings: P60: ‐1.5 mm: 0.0089; ‐0.9 mm: 0.0178; ‐0.3 mm: 0.0006; 0.3 mm: 0.0014; 1.5 mm: 0.0417; 2.1 mm: 0.0008; 2.7 mm: 0.0018. P90: ‐3 mm: 0.0316; ‐2.7 mm: 0.0113; ‐2.1 mm: <0.0001; ‐1.5 mm: <0.0001; ‐0.9 mm: 0.0004; ‐0.3 mm: <0.0001; 0.3 mm: <0.0001; 0.9 mm: 0.0004; 1.5 mm: <0.0001; 2.1 mm: <0.0001; 2.7 mm: 0,0007. P‐values determined by two‐way ANOVA with Sidak's post hoc comparison. Abbreviations: OS: outer segment; ONL: outer nuclear layer; INL: inner nuclear layer; ONH: optic nerve head; I: inferior; S: superior.

After confirming the cone‐specific knockout of STX3 in *Stx3^f/f(HRGP‐Cre)^* retina, we performed full‐field electroretinogram (ERG) to determine the functional impact of STX3 depletion. Scotopic ERGs revealed a significant reduction in the a‐wave responses at P60 (23.6% reduction) and further decreased at P90 (28.9% reduction, the last timepoint evaluated), indicating an impaired rod photoreceptor function in the *Stx3^f/f(HRGP‐Cre)^* retina (**Figure** **2**A,C). The decline in the scotopic a‐wave was preceded by a significant reduction in the scotopic b‐wave observed at P30 (23.4% reduction), worsening at P60 (31.2% reduction), and further declining at P90 (38.6% reduction, Figure 2A,C). Photopic ERGs showed a complete absence of cone function in *Stx3^f/f(HRGP‐Cre)^* retinas at all measured ages (Figure 2B,C). To rule out the possibility that the observed retinal dysfunction was caused by HRGP‐Cre expression itself, we performed ERG analysis on P60 mice expressing HRGP‐Cre but lacking Lox/P sites. No significant changes were observed in the scotopic a and b‐waves or photopic b‐waves (Figure S1A–C, Supporting Information).

To determine whether the observed functional decline is a consequence of photoreceptor cell loss, we performed morphometric analysis on retinal sections taken through the optical nerve at P30, P60, and P90 (Figure 2D–G). At P30, the *Stx3^f/f(HRGP‐Cre)^* retina showed no significant change in the outer nuclear layer (ONL) (Figure 2D‐left panels and E). However, by P60, a notable reduction in ONL nuclei was observed (Figure 2D‐middle panels and F), with further progression by P90 (Figure 2D‐right panels and G). In addition to the ONL count, we also assessed the overall thickness of the ONL at each time point (Figure S1D–F, Supporting Information). Consistent with the nuclear count, a significant decrease in ONL thickness was first evident at P60 and continued through P90 (Figure S1E,F, Supporting Information). Given that cone photoreceptors make up 3% of the mouse retina, and assuming complete degeneration of these cells, the observed loss of nuclei from the ONL at P60 (ranging from a 11.5% to 19.1% reduction, depending on the distance from the optic nerve head, ONH) and at P90 (ranging from a 12.5% to 27.3% reduction) was unexpected.^[^ ^45^ ^]^ Combined with the functional data, these findings suggest that STX3 depletion in cones not only affects cone photoreceptor integrity but also compromises rod health and function.

**Figure 3:**
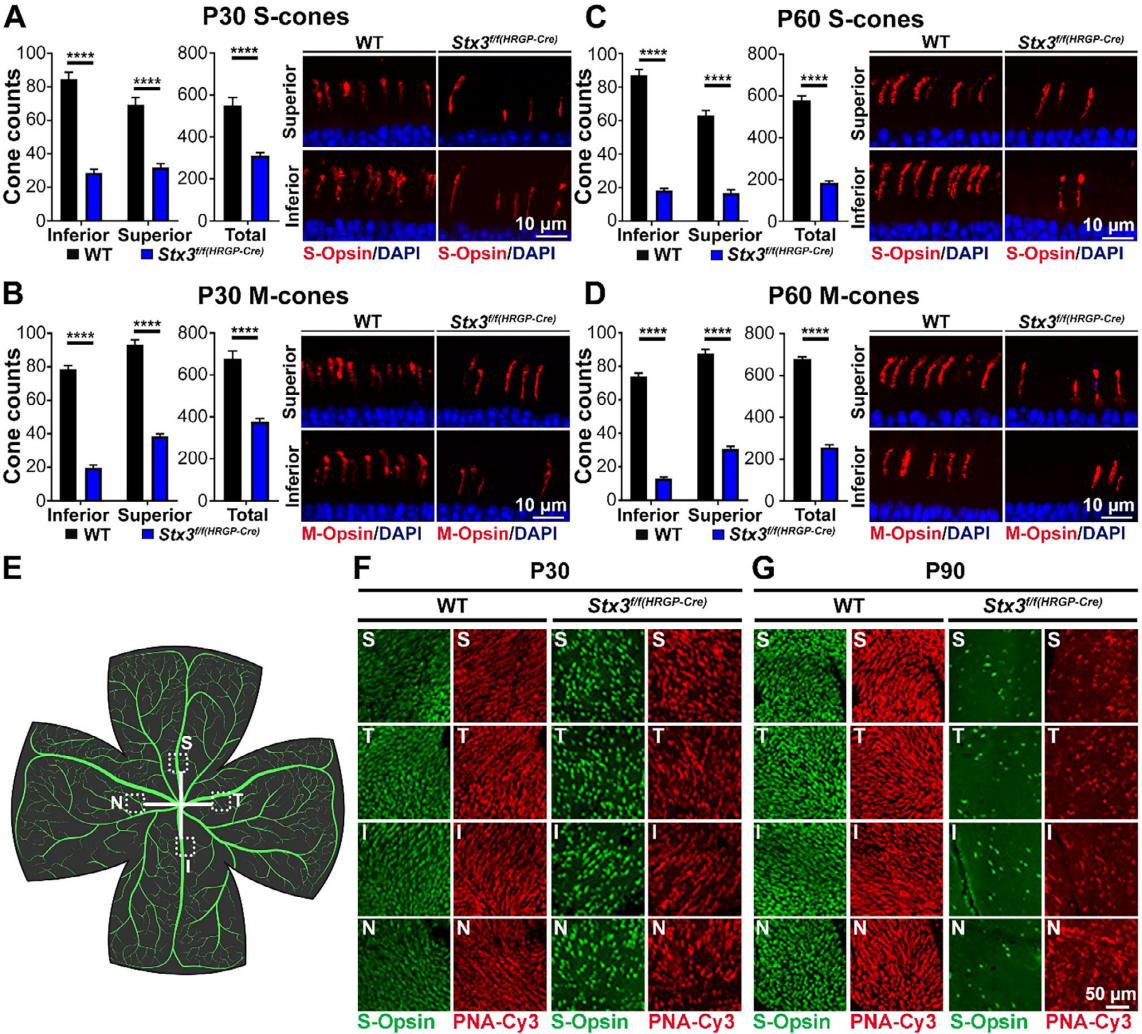
S‐ and M‐cones are lost in the *Stx3^f/f(HRGP‐Cre)^* retina. A‐D) Quantification of S‐cones (A, C; P30 and P60, respectively) and M‐cones (B, D; P30 and P60, respectively). Counts are shown for inferior and superior regions of the retina (left panels), total cone counts across the entire meridian (middle panels), and representative images (right panels). Data are presented as mean ± SEM. Significant reductions in cone numbers were observed in *Stx3^f/f(HRGP‐Cre)^* retinas. N‐values: P30 S‐cones: WT: 15 images, 4 mice; *Stx3^f/f(HRGP‐Cre)^*: 10 images, 3 mice. P30 M‐cones: WT: 16 images, 4 mice; *Stx3^f/f(HRGP‐Cre)^*: 10 images, 3 mice. P60 S‐cones: WT: 14 images, 4 mice; *Stx3^f/f(HRGP‐Cre)^*: 10 images, 3 mice. P60 M‐cones: WT: 14 images, 4 mice; *Stx3^f/f(HRGP‐Cre)^*: 12 images, 3 mice. P30 total S‐cones: WT: 11 images, 3 mice; *Stx3^f/f(HRGP‐Cre)^*: 14 images, 4 mice. P30 total M‐cones: WT: 12 images, 3 mice; *Stx3^f/f(HRGP‐Cre)^*: 14 images, 4 mice. P60 total S‐cones: WT: 15 images, 4 mice; *Stx3^f/f(HRGP‐Cre)^*: 10 images, 3 mice. P60 total M‐cones: WT: 15 images, 4 mice; *Stx3^f/f(HRGP‐Cre)^*: 12 images, 3 mice. P‐values for significant findings: All comparisons: <0.0001. P‐values determined by two‐way ANOVA with Sidak's post hoc comparison. E‐G) Retinal wholemounts were analyzed in the regions highlighted in (E). F) WT and *Stx3^f/f(HRGP‐Cre)^* retinas at P30, labelled for S‐Opsin (S‐cones) and PNA‐Cy3 (total cones), showing reduced S‐cones and total cones in the *Stx3^f/f(HRGP‐Cre)^* retinas. G) Wholemounts of WT and *Stx3^f/f(HRGP‐Cre)^* retinas at P90, revealing further cone loss in *Stx3^f/f(HRGP‐Cre)^* retinas. Abbreviations: S: superior; T: temporal; I: inferior; N: nasal. The scheme of wholemount in Figure 3E was created with BioRender.com.

Cone‐specific STX3 knockout leads to a reduction in cone photoreceptors, preceded by structural defects in cone connecting cilia. Given that STX3 depletion in the *Stx3^f/f(CRX‐Cre)^* retina results in photoreceptor cell death,^[^ ^27^ ^]^ we next quantified cone cell numbers in *Stx3^f/f(HRGP‐Cre)^* retina. Retinal sections from P30 and P60 mice were immunolabeled for either S‐ or M‐opsins. Cone cells were counted in a 350 µm^2^ area, located 300 µm from the optic nerve center, in both the central inferior and superior regions. At P30, cones labeled for S‐opsin were reduced by 66.2% while M‐opsin cones were reduced by 75.1% in the central inferior region. In the central superior region, cones labeled for S‐opsin decreased by 54.3% and those for M‐opsin were reduced by 58.9% (**Figure** **3**A,B). At P60, loss of cones progressed to 79.1% of S‐cones in the central inferior and 73.3% in the central superior regions, whereas M‐cones were reduced by 82.4% and 65.1%, respectively (Figure 3C,D). Total cone loss across the entire meridian was 43.5% for S‐cones at P30, increasing to 68.1% by P60, while loss of M‐cones was 44.2% at P30 and 62.2% at P60 (Figure 3A–D). While S‐cones were equally affected in the inferior and superior regions, loss of M‐cones was more pronounced in the inferior region at both time points. To assess whether STX3 depletion affects the nasal and temporal regions differently, we co‐stained retina wholemounts with S‐opsin and PNA‐Cy3 (Figure 3E–G; Figure S2, Supporting Information). The whole mounts revealed cone depletion at P30 (Figure 3F; Figure S2A,B, Supporting Information), which progressed through P90 (Figure 3G; Figure S2C,D, Supporting Information). However, no clear trend was observed in the extent of cone loss between the nasal and temporal regions.

**Figure 4:**
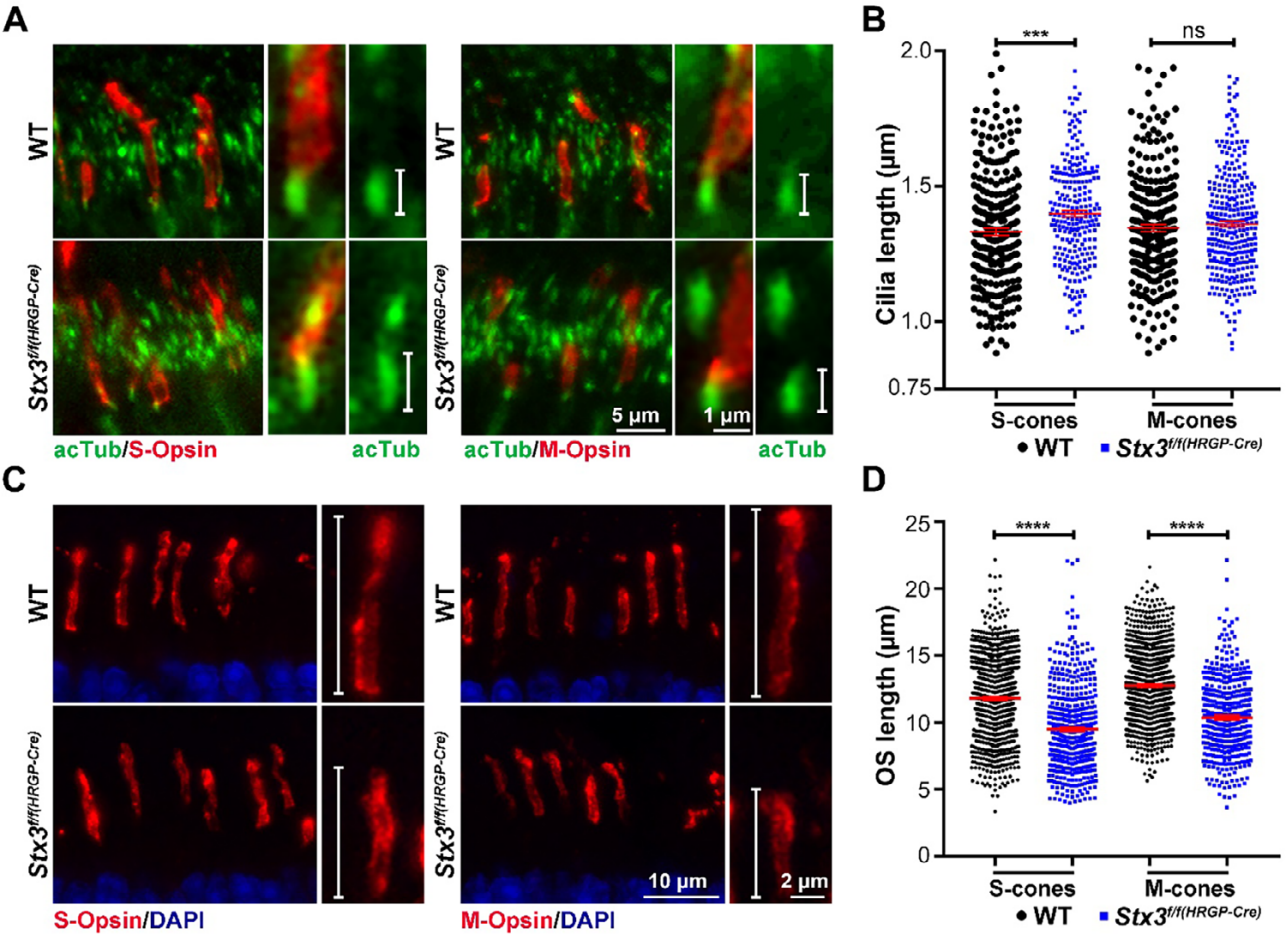
Structural anomalies in the connecting cilium and shortening of the outer segments of *Stx3^f/f(HRGP‐Cre)^* cone photoreceptors. A) Representative images of cone cilia in P30 W and *Stx3^f/f(HRGP‐Cre)^* retinas. B) Quantification of S‐cones, M‐cones, and total cones revealed a significant increase in ciliary length, specifically in S‐cones. N‐Values: S‐cones: 266 and 250 cilia for WT and *Stx3^f/f(HRGP‐Cre)^*, respectively; M‐cones: 247 and 318 cilia for WT and *Stx3^f/f(HRGP‐Cre)^*, respectively. Quantification is presented as mean ± SEM. P‐values for significant findings: Ciliary length of S‐cones: 0.0002. P‐values determined by a two‐tailed unpaired t‐test. C) Representative images displaying OS of S‐ and M‐cones in P30 WT and *Stx3^f/f(HRGP‐Cre)^* mice. D) Quantification of S‐ and M‐cone OS length in P30 WT and *Stx3^f/f(HRGP‐Cre)^* mice showing a significant shortening of both S‐ and M‐cone OS. N‐values: P30 S‐cones: WT: 1288 OS, 3 mice; *Stx3^f/f(HRGP‐Cre)^*: 515 OS, 3 mice. P30 M‐cones: WT: 1338 OS, 3 mice; *Stx3^f/f(HRGP‐Cre)^*: 451 OS, 3 mice. P‐values for significant findings: OS length of S‐cones: <0.0001. OS length of M‐cones: <0.0001. P‐values determined by a two‐tailed unpaired t‐test.

Photoreceptor cell death is often preceded by structural changes in the connecting cilium (CC), the narrow bridge linking the OS and IS of the photoreceptor cell.^[^ ^47^, ^48^, ^49^ ^]^ To assess potential structural alterations in the CC, we labelled sections of P30 WT and *Stx3^f/f(HRGP‐Cre)^* retinas for acetylated tubulin (which marks acetylated microtubules in the photoreceptor cilium) and for S‐opsin or M‐opsins, independently. Quantification of ciliary length revealed a significant increase in *Stx3^f/f(HRGP‐Cre)^* S‐opsin labeled cones (1.4 vs 1.33 µm for *Stx3^f/f(HRGP‐Cre)^* and WT mice, respectively), while M‐opsin labeled cones showed no significant difference (1.36 vs 1.35 µm for *Stx3^f/f(HRGP‐Cre)^* and WT mice, respectively) (**Figure** **4**A,B). In addition to the ciliary length, we also assessed the length of the cone OS in the *Stx3^f/f(HRGP‐Cre)^* model at P30 (Figure 4C,D). The OS of S‐cones (9.50 vs 11.82 µm, for *Stx3^f/f(HRGP‐Cre)^* and WT mice, respectively, Figure 4C) and M‐cones (10.37 vs 12.78 µm, for *Stx3^f/f(HRGP‐Cre)^* and WT mice, respectively, Figure 4D) were found to be significantly shortened in the *Stx3^f/f(HRGP‐Cre)^* at P30.

### 2.3 Cone‐Specific Knockout of STX3 Leads to STXBP1 Depletion

**Figure 5:**
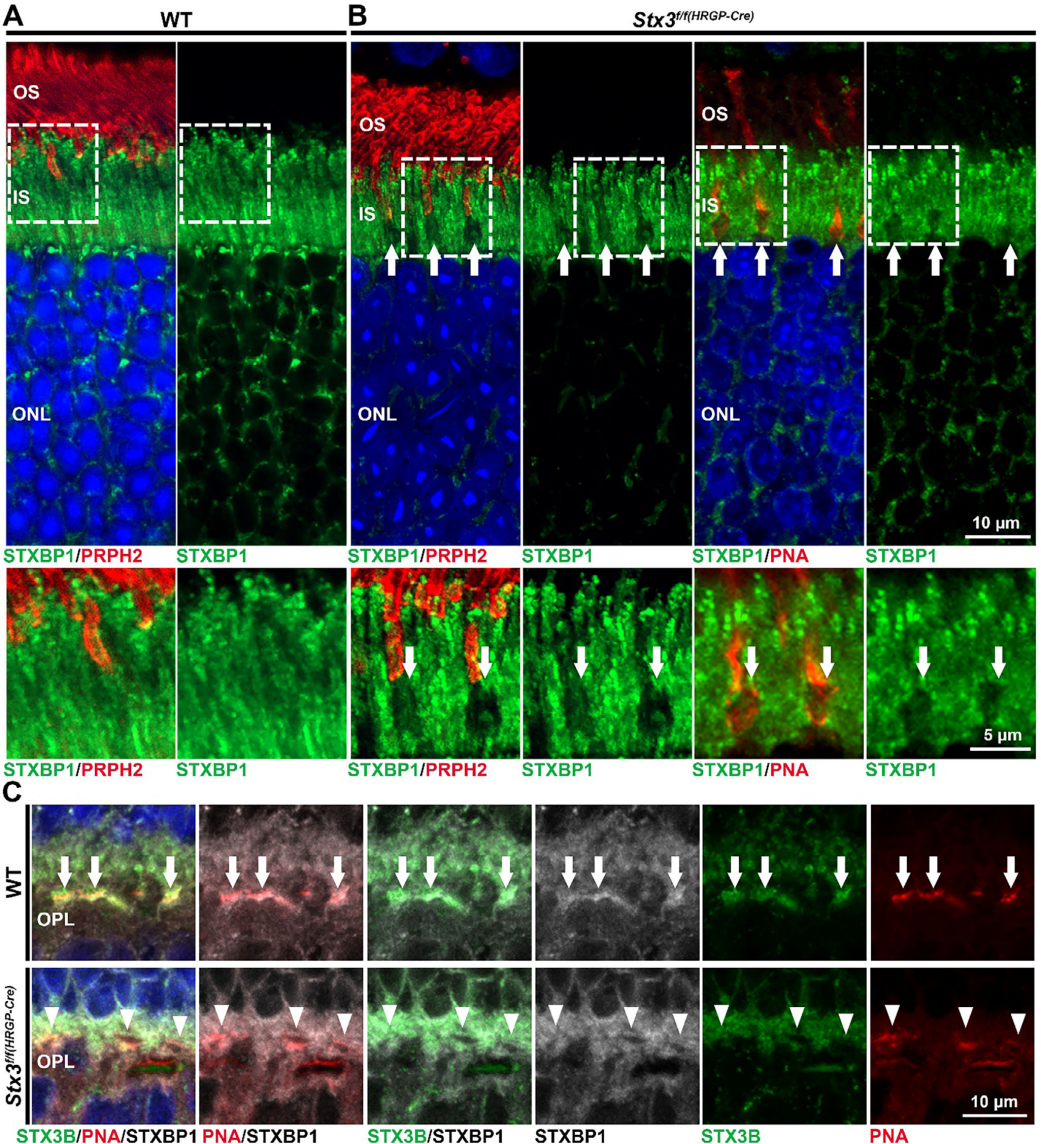
STXBP1 is co‐depleted with STX3 in the cones of the *Stx3^f/f(HRGP‐Cre)^* retina. A) Immunofluorescence images showing STXBP1 labeling in the WT retina at P30, co‐stained for PRPH2, which is localized to the OS of rods and cones. STXBP1 was localized in the IS of the retina. B) Co‐labeling for STXBP1, PRPH2 (left panel), and peanut agglutinin (PNA, right panel) reveals depletion of STXBP1 specifically in the OS of *Stx3^f/f(HRGP‐Cre)^* mice (arrows). C) Co‐labeling for STXBP1, PNA, and STX3B demonstrates a depletion of both STX3B and STXBP1 in the cone pedicles (labeled with PNA, arrowheads) in the *Stx3^f/f(HRGP‐Cre)^* retina, while a strong localization of STXBP1 can be observed at the cone pedicles in the WT mice (arrows). Abbreviations: OS: outer segment; IS: inner segment; ONL: outer nuclear layer; OPL: outer plexiform layer.

Since STXBP1 was found to interact with STX3,^[^ ^27^, ^43^ ^]^ we explored how STX3 depletion in cones impacts this interaction in *Stx3^f/f(HRGP‐Cre)^* retinas. Retinal sections from P30 WT and *Stx3^f/f(HRGP‐Cre)^* mice were co‐labeled for PRPH2 and STXBP1. In the WT retina, STXBP1 was confined to the IS and ONL (**Figure** **5**A). In the *Stx3^f/f(HRGP‐Cre)^* retina, however, no apparent mislocalization of SXTBP1 was detected (Figure 5B). Instead, gaps (suspected to be cone photoreceptors) in STXBP1 labeling were observed in the IS region. To determine the type of photoreceptor, we co‐labeled for STXBP1 and PNA, a marker for the cone glycocalyx. We found that the gaps in STXBP1 labeling consistently co‐localized with PNA staining, confirming that STXBP1 is specifically depleted in the ISs of cones in the *Stx3^f/f(HRGP‐Cre)^* retina (white arrows, Figure 5B). Since STXBP1 is known to localize at the photoreceptor synapse, we next examined STXBP1 labeling at cone pedicles, again marked with PNA. While STXBP1 was broadly distributed throughout the OPL in the WT retina, stronger labeling was seen around the cone pedicles (Figure 5C‐upper panels, white arrows). In contrast, STXBP1 labeling was absent from the cone pedicles in the *Stx3^f/f(HRGP‐Cre)^* retina (Figure 5C‐lower panels, arrowheads). These findings demonstrate that cone‐specific depletion of STX3 in *Stx3^f/f(HRGP‐Cre)^* mice leads to a loss of STXBP1 from both cone IS and synapses. This is in contrast to the fate of STXBP1 upon the depletion of STX3 in rods in the *Stx3^f/f(CRX‐Cre)^* retina, whereby the protein persisted.^[^ ^27^ ^]^ However, unlike the rods in the *Stx3^f/f(CRX‐Cre)^* retina,^[^ ^27^ ^]^ neither cone opsins nor PRPH2 and ROM1 were mislocalized in the *Stx3^f/f(HRGP‐Cre)^* retinas (Figure S3A,B, Supporting Information).

### 2.4 Cone‐Specific Depletion of STX3 Leads to Downregulation of ARR4

**Figure 6:**
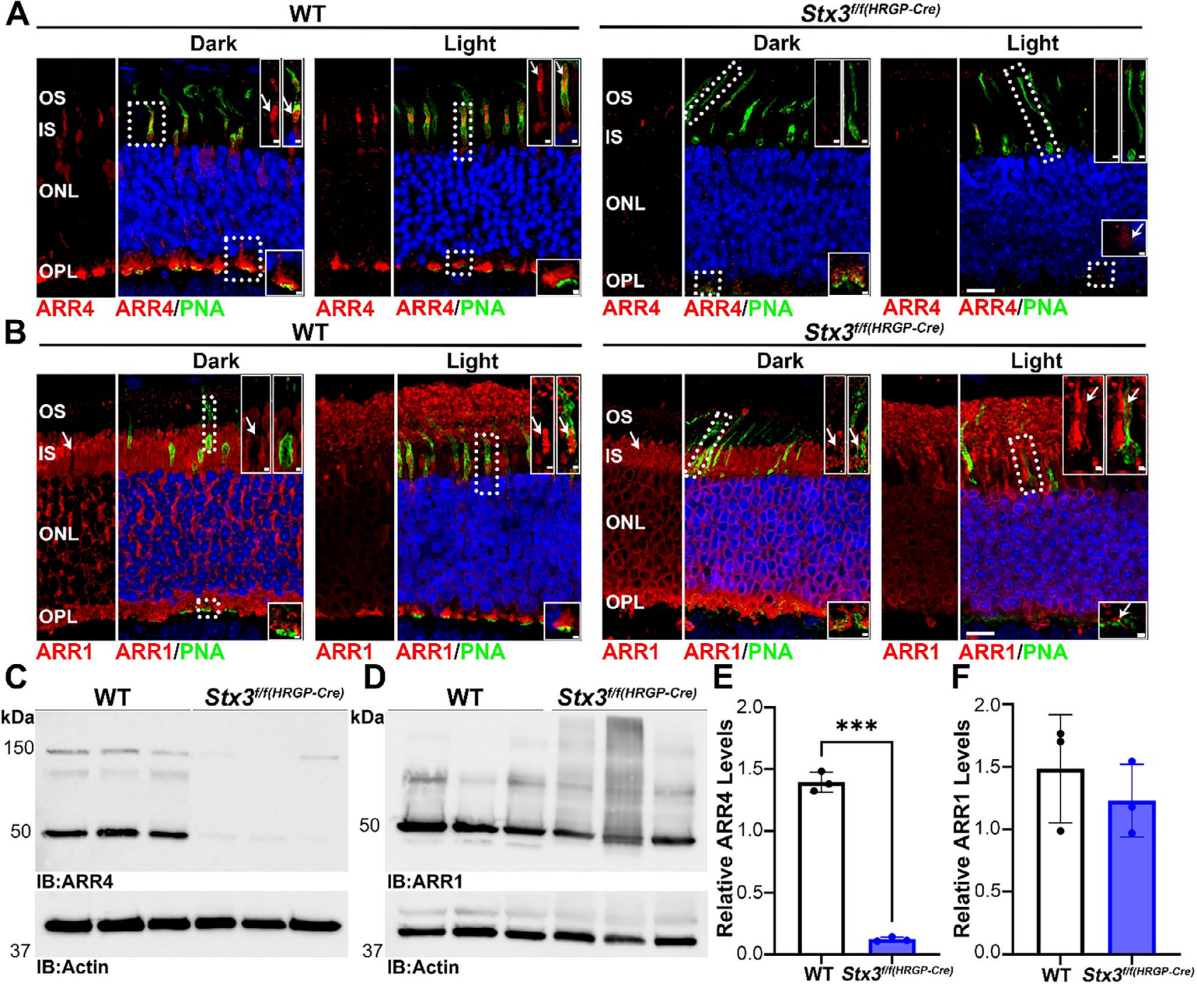
ARR4 is absent from the cones of the *Stx3^f/f(HRGP‐Cre)^* mice. A) Immunofluorescence labeling of light‐ and dark‐adapted P30 WT and *Stx3^f/f(HRGP‐Cre)^* retinas with PNA and ARR4. In dark‐adapted mice, ARR4 is located in the IS, while under light‐adapted conditions, ARR4 translocated to the OS (arrows). ARR4 labeling is depleted in both light‐ and dark‐adapted *Stx3^f/f(HRGP‐Cre)^* retinas (faint remaining ARR4 signal in OPL highlighted by arrow). B) Labeling with PNA and for ARR1 in both light‐ and dart‐adapted P30 WT and *Stx3^f/f(HRGP‐Cre)^* mice (ARR1 localization IS, OS, and cone pedicles highlighted by arrows). C & D) Immunoblot analysis of three independent WT and *Stx3^f/f(HRGP‐Cre)^* retinal samples, probed with antibody against ARR4 (C) and ARR1 (D), with actin as a loading control. (Note: The immunoblot in Figure 1C was re‐probed, re‐imaged, and re‐used for D.) Almost complete depletion of ARR4 could be observed in the *Stx3^f/f(HRGP‐Cre)^* samples. Minor changes in ARR1 expression levels between WT and *Stx3^f/f(HRGP‐Cre)^* mice were observed. E & F) Quantification of the immunoblot data from (C, D) confirms the near‐complete depletion of ARR4 and a non‐significant reduction in ARR1. Quantification is presented as mean ± SD. N‐Value: 3 for WT and *Stx3^f/f(HRGP‐Cre)^*. P‐values for significant findings: relative ARR4 levels in E: 0.0009. P‐values determined using a two‐tailed unpaired t‐test with Welch's correction.

In this study, we initially used cone‐arrestin (ARR4) as a cone‐specific marker to assess cone health. However, we observed a significant reduction in its signal in retinal sections from *Stx3^f/f(HRGP‐Cre)^* mice, prompting further investigation. ARR4 translocates from the cone IS to the OS following light exposure.^[^ ^50^ ^]^ To determine whether ARR4 expression or translocation is altered in *Stx3^f/f(HRGP‐Cre)^* mice, we exposed both *Stx3^f/f(HRGP‐Cre)^* and WT mice at P30 to light‐ and dark‐adapted conditions and co‐labeled retinal sections with PNA and ARR4 (**Figure** **6**A). In the dark‐adapted WT retina, ARR4 was localized to the cone cell bodies, ISs (see white arrows in upper insets), as well as the cone pedicles in the OPL (see bottom inset and dotted box). There is also a signal detected in the OSs. After light exposure, most ARR4 translocated from the cone IS to OS, presented by white arrows in the upper inset (selected cell is denoted by the dotted box), while labeling in the synaptic terminals was maintained (bottom inset). In the *Stx3^f/f(HRGP‐Cre)^* retina, cone‐specific depletion of STX3 led to a dramatic reduction of ARR4 in cones (Figure 6A, right). In both light‐ and dark‐adapted *Stx3^f/f(HRGP‐Cre)^* retinas, the ARR4 signal was barely detectable in the OS or IS of the cones (see upper insets in both light and dark conditions). Faint ARR4 labeling was observed at the cone pedicles in dark‐adapted retinas, but was absent in light‐adapted *Stx3^f/f(HRGP‐Cre)^* retinas (Figure 6A, right, see bottom insets). To confirm the association between ARR4 expression and that of STX3, we analyzed ARR4 levels in heterozygous knockout animals (*Stx3^f/+(HRGP‐Cre)^*). Immunofluorescence (IF) labeling showed ARR4 localization at the cone OS and synapses in the heterozygotes (Figure S4A, Supporting Information). Both STX3B and ARR4 expression levels in *Stx3^f/+(HRGP‐Cre)^* retinas were comparable to those in WT controls (Figure S4B,C, Supporting Information).

While rod photoreceptors express only Arrestin 1 (ARR1), cones express both ARR1 and ARR4.^[^ ^51^ ^]^ Similar to ARR4, ARR1 translocates to the OS upon light exposure.^[^ ^50^ ^]^ In dark‐adapted WT retina (Figure 6B, left), ARR1 localizes to both rod and cone synaptic terminals (see bottom inset), perinuclearly, and to the ISs. In the PNA‐labeled cone IS, ARR1 is notably absent (see white arrows and upper inset). After light exposure, while ARR1 is maintained in the cone pedicle (lower inset), most translocate to the cone IS (white arrows, upper inset). In light‐exposed rods, the majority of ARR1 labeling is found in the OS. Dark‐adapted *Stx3^f/f(HRGP‐Cre)^* rods maintained the localization patterns of WT, with substantial labeling in the OPL, ONL, and IS of rods. Resembling the WT, ARR1 was also absent in the cone IS (upper inset) and was present in the IS after light exposure (Figure 6B, right, white arrows, upper insets). This observation is further supported by Figure S5 (Supporting Information), where confocal images and 3D reconstructions further confirmed ARR1 localization in the cone ISs under light‐adapted conditions in both WT and *Stx3^f/f(HRGP‐Cre)^*. Bottom insets show that abnormal *Stx3^f/f(HRGP‐Cre)^* cone synapses maintain a trace amount of ARR1 labeling.

To confirm the downregulation of ARR4 and evaluate whether there are any changes in ARR1 levels in the *Stx3^f/f(HRGP‐Cre)^* retina, we performed IB with three independent samples from P30 WT and *Stx3^f/f(HRGP‐Cre)^* mice. The ARR4 band at ≈50 kDa was nearly absent in the *Stx3^f/f(HRGP‐Cre)^* retina (Figure 6C), while ARR1 expression in *Stx3^f/f(HRGP‐Cre)^* retina showed a minor difference from WT (Figure 6D, reprobed blot from Figure 1D). Quantification of IB revealed an almost complete loss of ARR4 in the *Stx3^f/f(HRGP‐Cre)^* retina, with a 91.1% reduction (Figure 6E), confirming that cone‐specific depletion of STX3 leads to ARR4 depletion. In contrast, ARR1 levels showed only a slight, statistically insignificant reduction (17.2%) in the *Stx3^f/f(HRGP‐Cre)^* retina (Figure 6F). These findings demonstrate that STX3 depletion differentially affects ARR1 and ARR4 isoforms, further highlighting the distinct roles of STX3 in cones versus rods.

### 2.5 Differential Requirement of STX3 for ARR1 and ARR4 Expression in Rods and Cones

**Figure 7:**
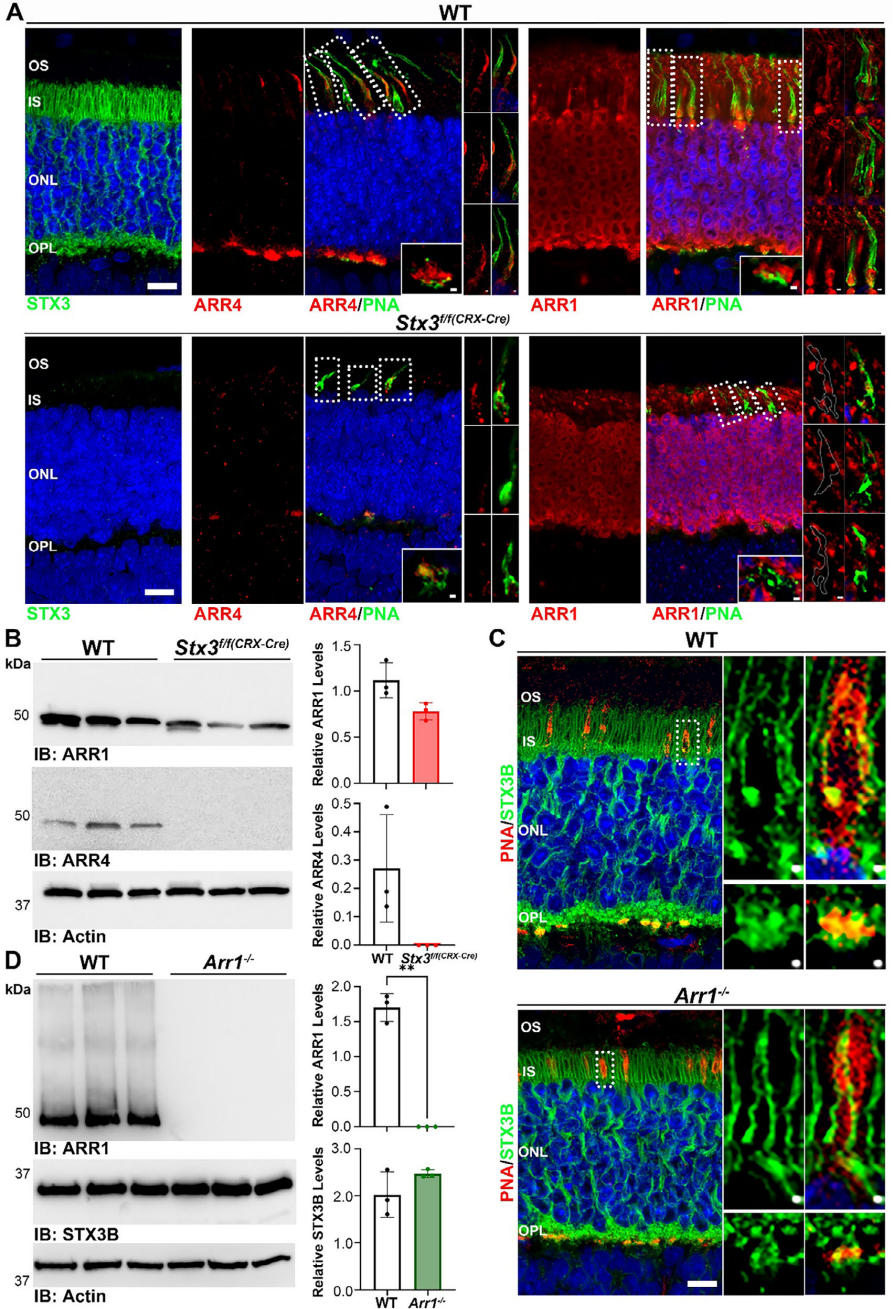
ARR1 and STX3 expression are not co‐dependent. A) Immunofluorescence labeling of P15 WT and *Stx3^f/f(CRX‐Cre)^* retinal sections from mice reared in cyclic light with PNA and for either ARR4 or ARR1. The signal of ARR1 and ARR4 was clearly reduced in *Stx3^f/f(CRX‐Cre)^* retinas. B) Immunoblot of three independent P15 WT and *Stx3^f/f(CRX‐Cre)^* samples probed with ARR1 and ARR4 antibodies. Actin was used as a loading control. Quantification of the immunoblot (right) shows no significant reduction of ARR1 levels and complete depletion of ARR4 in *Stx3^f/f(CRX‐Cre)^* retinas. C) Immunofluorescence of P30 WT and *Arr1^−/−^* retinas labeled with PNA and for STX3B. STX3B localization remains unchanged in the *Arr1^−/−^* retina. D) Immunoblot of three independent P30 WT and *Arr1^−/−^* retina samples probed for ARR1 and STX3B antibodies. Quantification of immunoblot (right) confirms the absence of ARR1 and shows no difference in STX3B expression between WT and *Arr1^−/−^* retinas. P‐values for significant findings: relative ARR4 levels: 0.0023. Quantification is presented as average ± SD. N‐value: 3 for P15 WT, P30 WT, P15 *Stx3^f/f(CRX‐Cre)^*, and P30 *Arr1^−/−^*. P‐values determined using a two‐tailed unpaired *t*‐test with Welch's correction.

To determine whether the downregulation of ARR4 and the maintenance of ARR1 were reproducible in another model of early onset cone‐rod STX3 elimination,^[^ ^27^ ^]^ we assessed the behavior of both isoforms in *Stx3^f/f(CRX‐Cre)^* mice. Retinal sections from P15 WT and *Stx3^f/f(CRX‐Cre)^* mice raised under cyclic light conditions were co‐labeled with PNA and ARR1 or ARR4 (**Figure** **7**A). P15 animals were selected to avoid the extensive degeneration that occurs after P21 in *Stx3^f/f(CRX‐Cre)^* retinas.^[^ ^27^ ^]^ In WT mice, ARR4 was located predominantly in synaptic terminals of cones; a faint signal could be observed in the IS, and a robust signal was observed in the OS (Figure 7A, top left). ARR1 was present in the OSs, ISs, and synaptic terminals of both rods and cones. Panels on the right of cells demarked by dotted boxes (Figure 7A, top right) further illustrate the IS and OS localizations of ARR1 and ARR4 under cyclic light conditions. In the *Stx3^f/f(CRX‐Cre)^* retina, ARR4 was largely depleted, whereas ARR1 was specifically absent in cones (Figure 7A, bottom panels). Outlines of selected cones (marked by white dotted boxes) show the lack of ARR1 within PNA‐labeled cones (Figure 7A, bottom right). Notably, this finding contrasts with observation in *Stx3^f/f(HRGP‐Cre)^* retinas, where ARR1 remained localized to the cone ISs under light‐adapted conditions. This discrepancy may be attributed to the embryonic onset of *Cre* expression in the *CRX‐Cre* model or the severe abnormalities in *Stx3^f/f(CRX‐Cre)^* cones, which could impact ARR1 localization.

Immunoblotting was performed to determine the extent of ARR1 and ARR4 depletion in the *Stx3^f/f(CRX‐Cre)^* retina compared to the WT controls (Figure 7B). ARR4 was completely absent in the *Stx3^f/f(CRX‐Cre)^* retina, while ARR1 showed a statistically insignificant reduction of ≈30.2%. Given the apparent loss of ARR1 and ARR4 in *Stx3^f/f(CRX‐Cre)^* cones, it was important to determine whether STX3 expression depended on arrestin expression. We aimed to evaluate both *Arr1^−/−^* and *Arr4^−/−^* retinas, however, only *Arr1^−/−^* animals were readily available (Figure 7C). In WT mice, STX3 prominently localized to the ISs of both rods and cones, as well as to the synapses of both photoreceptor types. Co‐labeling with PNA highlighted the localization of STX3 at cone ISs and synapses (Figure 7C). In the *Arr1^−/−^* retinas, no changes in STX3 localization were observed. IBs confirmed the absence of ARR1 in *Arr1^−/−^* retinas, while STX3 levels were insignificantly altered from WT (ratios of 2.02 and 2.48 of STX3 to actin for WT and *Arr1^−/−^*, respectively, Figure 7D). These results confirm that STX3 is essential for the presence of ARR4 in cones, while it appears expendable for the presence of ARR1 in rods. Furthermore, in *Stx3^f/f(CRX‐Cre)^* cones, the elimination of STX3 may affect ARR1 presence in cones, but ARR1 is not essential for the existence of STX3 in either rod or cone photoreceptors.

### 2.6 Light and Dark Modulation of STX3 Interactions with ARR4 and STXBP1

**Figure 8:**
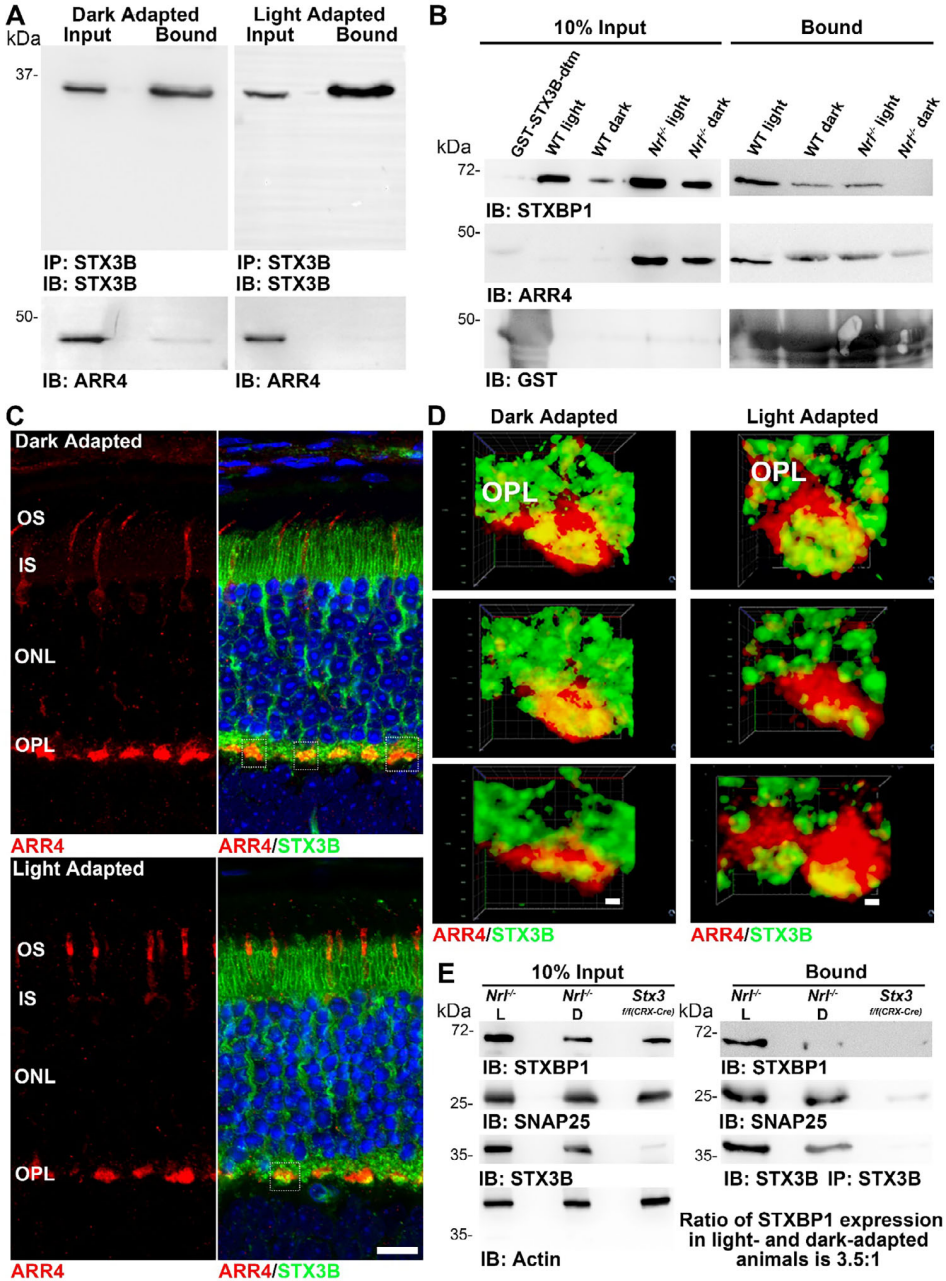
The interaction of STX3 with ARR4 and STXBP1 is modulated in light and dark conditions. A) Immunoprecipitation using retinal extracts from P30 WT mouse, either light‐ or dark‐adapted, using the anti‐STX3B antibody. ARR4 was immunoprecipitated with STX3B, with stronger binding of ARR4 to STX3B observed in dark‐adapted animals. B) GST‐pulldown using recombinant GST‐tagged STX3‐dtm (STX3 lacking the transmembrane domain) with retinal lysates from light‐ and dark‐adapted P30 WT and *Nrl^−/−^* mouse retinal extracts. ARR4 was immunoprecipitated from both genotypes under light and dark conditions. STXBP1 was also immunoprecipitated from both genotypes; however, no STXBP1 was brought down using lysates from dark‐adapted *Nrl^−/−^* mice. C) Immunofluorescence analysis of light‐ and dark‐adapted P30 WT retinas showing STX3B localization at cone pedicles. Under light adaptation, STX3B displayed a punctate distribution, whereas under dark adaptation, labeling appeared more diffuse and expanded. D) 3D modelling of three representative cone pedicles from light‐ and dark‐adapted WT animals. STX3B and ARR4 co‐localize at the cone pedicles under both light‐ and dark‐conditions. However, in dark‐adapted animals, STX3B labelling at the cone pedicles was more expanded. E) Immunoprecipitation using retinal extracts from P60 light‐ and dark‐adapted *Nrl^−/−^* retinas using anti‐STX3B antibody. Strong binding of STXBP1 to STX3B could be observed in the light‐adapted condition, while almost no binding could be observed in the dark‐adapted condition. The interaction with SNAP25 was not impacted by the light and dark exposure. Abbreviations: OS: outer segment; IS: inner segment; ONL: outer nuclear layer; OPL: outer plexiform layer; WT L and WT D: light‐ and dark‐adapted WT mice; *Nrl^−/−^* L and *Nrl^−/−^* D: light‐ and dark‐adapted *Nrl^−/−^* mice; *Stx3^f/f(CRX‐Cre)^*: *Stx3^f/f(CRX‐Cre)^* reared in cyclic light.

The depletion of ARR4 in cones of *Stx3^f/f(HRGP‐Cre)^* mice prompted further analysis of this interaction. To further investigate the light's effects on the STX3‐ARR4 interaction, we performed immunoprecipitation (IP) of STX3 from retinal lysates of light‐ and dark‐adapted P30 WT mice. We found that ARR4 interacts with STX3 primarily in dark‐exposed retinal lysates (**Figure** **8**A). To further characterize this interaction, we conducted a GST‐pulldown assay using bacterially expressed recombinant GST‐tagged STX3 lacking its transmembrane domain (GST‐STX3‐dtm). In addition to WT retinas, we used *Nrl^−/−^* retinas, which are cone‐like, to explore the cone‐specific properties of the interactions. Mice from both genotypes and light conditions were analyzed. Notably, ARR4 interacted with STX3 under all conditions and genotypes, with no significant changes in the strength of the interaction (Figure 8B).

To determine whether the light‐induced changes in the STX3‐ARR4 interaction are attributed to alterations in the subcellular localization of ARR4, we co‐stained for STX3 and ARR4 on retinal sections from light and dark‐adapted WT mice (Figure 8C,D). In dark‐adapted retinas, ARR4 was localized to the cone ISs, OSs, and cone pedicles within the OPL (Figure 8C,D). Under light‐adaption, ARR4 showed increased accumulation in the OS, while its localization in the OPL remained unchanged (Figure 8C,D). STX3 localization in the IS was unaffected by the light condition; however, light exposure induced distinct changes in its distribution within the OPL (Figure 8D). Specifically, STX3 displayed a diffuse pattern in the dark, becoming more punctate in the light‐adapted conditions. Despite these alterations, the extent of colocalization between ARR4 and STX3 at cone pedicles appeared largely consistent across light and dark conditions.

Given the cone‐specific depletion of STXBP1 in the retina of *Stx3^f/f(HRGP‐Cre)^* mice, we next examined the interaction between STX3 and STXBP1 in light‐ and dark‐adapted conditions using GST‐pulldown assays in WT and *Nrl^−/−^* retinas (Figure 8B). In WT retinas, the STX3‐STXBP1 interaction was unaffected by light or dark adaptation (Figure 8B). In contrast, in the cone‐like retinas of *Nrl^−/−^* mice, this interaction was lost under dark‐adapted conditions (Figure 8B). To further validate this finding, we performed IP from light‐ and dark‐adapted *Nrl^−/−^* retinas. STXBP1 was robustly co‐precipitated with STX3 in light‐adapted retinas, whereas markedly reduced amounts were detected in dark‐adapted samples (Figure 8E), confirming the GST‐pulldown results. Notably, the interaction between STX3 and SNAP25 was not affected by light‐ or dark‐adaption in the *Nrl^−^* ^/−^ mice.

## 3 Discussion

Syntaxins are essential components of SNARE complexes, orchestrating membrane fusion events for intracellular trafficking and synaptic transmission. In the retina, STX3, VAMP2, and SNAP25, mediate synaptic vesicle fusion at the ribbon synapses of both rod and cone photoreceptors.^[^ ^18^, ^19^, ^20^, ^23^ ^]^ While STX3's role in neurotransmitter release is well established, its function in the photoreceptor inner segment remains less defined.^[^ ^19^ ^]^ Prior studies have implicated a STX3, VAMP7, and SNAP25 complex in RHO trafficking from the IS to the OS.^[^ ^24^, ^25^ ^]^ In a previous rod‐ and cone‐specific STX3 knockout (*Stx3^f/f(CRX‐Cre)^*), we observed partial rhodopsin mislocalization, supporting the role of STX3 in OS protein trafficking.^[^ ^27^ ^]^ Although STX3 was depleted in cones of this model, we found cone opsins were properly localized.^[^ ^27^ ^]^ This observation clearly suggests divergent roles of STX3 in rods and cones. To explore STX3's function in cones, we generated a cone‐specific STX3 knockout (*Stx3^f/f(HRGP‐Cre)^*), which resulted in the complete loss of cone function, reduction in the number of both S‐ and M‐cones, and elongation of S‐cone connecting cilia. We identified ARR4 as a novel interactor of STX3, with a preferential interaction in dark‐adapted cones. Additionally, the regulatory protein STXBP1 was co‐depleted with STX3 in cones, an effect not observed upon STX3 elimination from rods, suggesting cell‐type‐specific regulation of STX3‐STXBP1 interaction (major findings summarized in Table 1).

**Table 1:** Summary of phenotypes and their effects on STXBP1 and ARR4 interaction in the *Stx3^f/f(HRGP‐Cre)^* model.

	WT	*Stx3^f/f(HRGP‐Cre)^*	
Cone function	Normal photopic ERG	No photopic b‐wave	
Rod function	Normal scotopic ERG	‐ Scotopic a‐wave is significantly decreased, beginning at P60. ‐ Scotopic b‐wave is significantly decreased, beginning at P30.	
Photoreceptor health overall	No photoreceptor loss	‐ Significant reduction in ONL nuclei count and thickness begins at P60. ‐ Reduction progresses through P90.	
Cone health	Normal	‐ Significant reduction in S‐ and M‐cones begins at P30. ‐ Progressive S‐ and M‐cone loss continues through P60.	
Cone structure	Normal	‐ Elongation of cilia occurs exclusively in S‐cones, beginning at P30. ‐ Structural disruption of cone synapses is observed starting at P30.	
Interactor	WT	*Stx3^f/f(HRGP‐Cre)^*	Role light vs dark
STXBP1	Present in the IS and photoreceptor synapse	Depleted from the cone IS and synapse	‐ Binding to STX3B occurs almost exclusively under light‐adapted conditions in the cone‐like retina. ‐ Light‐dependent interaction with STX3B modulates SNARE complex formation in cones
ARR4	Present in cone IS, OS (translocation in light), and synapse	Depleted from cone IS, OS and synapse	‐ Translocates toward cone OS after light exposure. ‐ Interacts with STX3B in dark‐adapted retina. ‐ ARR4/STX3B interaction increases vesicle release from cone ribbon synapses in dark‐adapted retinas (dark current).

Despite cones comprising only ≈3% of the total photoreceptor population, cone‐specific STX3 depletion reduced total retinal STX3 by 31.8%. This disproportionate reduction may reflect the structural and functional complexity of cone ribbon synapses, which contain up to 15 ribbons per synapse compared to a single ribbon in rods.^[^ ^52^, ^53^ ^]^ Studies in the salamander retina indicated that cone vesicle release is triggered by the activation of 2–3 Ca^2+^ channels, compared to 3–5 channels in rods.^[^ ^54^, ^55^ ^]^ Additionally, exocytosis occurs significantly faster in cones than in rods.^[^ ^56^ ^]^ This increased speed and lower Ca^2+^ threshold suggest that cone ribbon synapses face higher synaptic demands. To meet these demands, cone ribbons may express elevated levels of SNARE complex components, such as STX3, VAMP2, and SNAP25, relative to rod ribbons.

Functionally, the *Stx3^f/f(HRGP‐Cre)^* retina exhibited a complete absence of photopic b‐waves across all postnatal ages analyzed, consistent with disrupted cone neurotransmitter release. We also observed progressive reduction in scotopic a and b‐waves, beginning as early as P30 and becoming more pronounced by P60. Although rod and cone pathways were traditionally considered separate, increasing evidence supports crosstalk between photoreceptor subtypes and bipolar cells.^[^ ^57^, ^58^, ^59^, ^60^, ^61^, ^62^, ^63^, ^64^, ^65^, ^66^ ^]^ Thus, cone dysfunction in our current model may indirectly interfere with rod‐mediated signaling. The progressive decline in rod function, coupled with ONL thinning and rod photoreceptor loss, indicates that the *Stx3^f/f(HRGP‐Cre)^* retina models cone‐rod dystrophy (CRD), a condition where primary cone degeneration is followed by secondary rod loss.^[^ ^67^ ^]^ A similar pattern was observed in the *Mitf^mi/+^* CRD model, where cone functional decline preceded rod dysfunction.^[^ ^68^ ^]^ In our model, significant thinning of the ONL coincided with a marked reduction in the scotopic a‐wave. Although these changes reached statistical significance at P60, it is likely that rod function was already compromised prior to overt rod cell loss. This early functional decline may also underlie the pronounced reduction in the scotopic b‐wave observed as early as P30 in the *Stx3^f/f(HRGP‐Cre)^* retina.

Cone photoreceptor numbers were significantly reduced at P30, with S‐cones and M‐cones showing a 43.5% and 44.2% loss, respectively. The critical role of efficient neurotransmitter vesicle release in photoreceptor survival was previously demonstrated in our *Stx3^f/f(CRX‐Cre)^* model, where pan‐photoreceptor deletion of STX3 led to cell death as early as P15.^[^ ^27^ ^]^ Additional models support this link between synaptic vesicle release and photoreceptor viability. For example, conditional knockout of SNAP25 in photoreceptors using the *Crx* promoter disrupted the SNARE complex formation, resulting in complete loss of visual function and subsequent photoreceptor degeneration.^[^ ^69^ ^]^ Similarly, in an autoimmune encephalomyelitis model, impaired exocytotic of neurotransmitter vesicles was associated with visual deficits and eventual optic nerve demyelination.^[^ ^70^ ^]^ In the *Stx3^f/f(HRGP‐Cre)^* model, cone degeneration continued with age: by P60, S‐cones were reduced by 68.1% and M‐cones by 62.2%. Notably, cone loss was more pronounced in the central inferior retina at both time points analyzed, likely due to greater cumulative light exposure, which is known to exacerbate cone degeneration in this region of the retina.

Another notable finding was the significant elongation of connecting cilia in S‐cones at P30. Ciliary elongation has previously been associated with photoreceptor cell death, as observed in male germ cell‐associated kinase knockout mice,^[^ ^47^ ^]^ implicating dysregulated ciliary length control in photoreceptor degeneration. In the *Stx3^f/f(HRGP‐Cre)^* retina, this elongation occurred at a stage when approximately half of the S‐ and M‐cones had already degenerated. Interestingly, this contrasts with our earlier *Stx3^f/f(CRX‐Cre)^* study, in which STX3 loss resulted in a significant reduction in ciliary length.^[^ ^27^ ^]^ This discrepancy may stem from methodological differences: the previous analysis did not distinguish between rod and cone cilia, and given that cones constitute only ≈ 3% of total photoreceptors in the mouse retina, the majority of cilia assessed were likely rod‐derived. The current study specifically examines cone cilia, suggesting that STX3 deficiency differentially affects rod and cone cilia. Moreover, ciliary elongation was not observed in M‐cones, suggesting subtype‐specific regulation of ciliary architecture. Evolutionarily, mammalian rods are thought to have evolved from S‐cones, and as a result, S‐cones and rods share a subset of phototransduction components, distinct from those in M‐ and L‐cones (e.g., primate retina).^[^ ^71^, ^72^ ^]^ The depletion of STX3 in cones may disrupt the transport of phototransduction components more severely in S‐cones than in M‐cones. Despite these structural differences, both S‐ and M‐cones displayed a significant shortening of the OS in the *Stx3^f/f(HRGP‐Cre)^* retina, and ultimately degenerate at a comparable rate.

STXBP1 was markedly depleted in cone OS and synaptic terminals in the *Stx3^f/f(HRGP‐Cre)^* retina, but not in rods of *Stx3^f/f(CRX‐Cre)^* mice, indicating a greater dependency on STX3 in cones. While traditionally viewed as a negative regulator of STX1‐mediated vesicle release,^[^ ^35^, ^36^, ^37^ ^]^ more recent work shows STXBP1 is also critical for SNARE complex assembly and vesicle priming.^[^ ^38^, ^39^, ^40^, ^41^, ^42^ ^]^ In the retina, STXBP1 localizes to the IS and the OPL, where it interacts with STX3.^[^ ^27^, ^43^ ^]^ This interaction is modulated by STX3 phosphorylation at tyrosine 14 by CaMKII under dark conditions, enhancing SNARE complex formation and vesicle release.^[^ ^73^, ^74^ ^]^ However, the STX3‐STXBP1 relationship is not linear. For example, a patient‐derived STXBP1 mutation (c.47A>G) increases binding to STX3 but paradoxically reduces vesicle release,^[^ ^75^ ^]^ suggesting that timely binding and release of STXBP1 from the SNARE complex is critical for function. Our findings support this model and indicate photoreceptor‐type‐specific regulation of STXBP1‐STX3 interaction. In cones, STXBP1 and STX3 were co‐depleted, a result not observed in rods,^[^ ^27^ ^]^ and their interaction was disrupted in dark‐adapted *Nrl^−/−^* retinas (which contain only cone‐like photoreceptors). This disruption was confirmed by immunoprecipitation and GST pulldown, although a limitation of the latter is the use of bacterially expressed STX3, lacking endogenous posttranslational modifications. These findings suggest that cone‐specific modifications, such as phosphorylation of STXBP1, may regulate its interaction with STX3. Notably, phosphorylation of STXBP1 is known to reduce its affinity to STX1A in conventional synapses.^[^ ^76^, ^77^ ^]^ While this disruption could imply that STXBP1 dissociation in cones enhances vesicle release in darkness (to support the dark current), this mechanism appears exclusive to cones, as STX3/STXBP1 interaction in WT rod‐dominant retinas remained unaffected by light conditions. Moreover, STXBP1 likely plays a facilitative, not purely inhibitory, role in vesicle release.^[^ ^35^, ^36^, ^37^, ^38^, ^39^, ^40^, ^41^, ^42^ ^]^ Together, these results point to distinct regulatory mechanisms for STXBP1 in rods versus cones. Future work should examine how posttranslational modifications of STXBP1, such as phosphorylation, differ between photoreceptor types and how this impacts light‐dependent synaptic transmission.

In this study, we identified cone‐specific interaction between STX3 and ARR4, a cone‐exclusive arrestin.^[^ ^50^ ^]^ Although ARR1 contributes ≈98% of total arrestin in mouse cones and ARR4 only 2%,^[^ ^51^ ^]^ both *Arr1^−/−^* and *Arr4^−/−^* mice show similar delays in cone response shutoff, suggesting ARR4 may have a higher affinity for cone opsins.^[^ ^51^ ^]^ In the dark, both ARR1 and ARR4 localize to the IS and synapse of cones,^[^ ^50^ ^]^ and both translocate to the OS upon light exposure, although some remain at the synapse. This light‐driven translocation was observed in WT mice, but in *Stx3^f/f(HRGP‐Cre)^* retina, ARR4 was nearly absent under both lighting conditions, whereas ARR1 localization and light‐dependent translocation were preserved. Immunoblotting confirmed a specific loss of ARR4 protein in *Stx3^f/f(HRGP‐Cre)^* cones, while ARR1 levels remained unchanged. In contrast, *Arr1^−/−^* retinas retained normal STX3 expression, indicating that ARR1 and STX3 do not depend on each other's stability or localization. Based on these findings, we focused further analysis on ARR4. In the *Stx3^f/f(CRX‐Cre)^* mice, where STX3 is depleted in both rods and cones, ARR4 was also absent in cones, reinforcing that ARR4 expression requires STX3. Given that STX3 is phosphorylated at T14 under light conditions and that ARR4 translocates in response to light, we investigated whether this interaction is light‐regulated. Co‐immunoprecipitation revealed a stronger STX3‐ARR4 interaction in dark‐exposed retinas. Since GST‐STX3‐dtm pulls down ARR4 from both light‐ and dark‐adapted lysates, we proposed that a light‐dependent modification, such as dephosphorylation at T14,^[^ ^73^, ^74^ ^]^ reduces STX3‐ARR4 interaction in vivo. Importantly, ARR4 co‐localized with STX3 at cone synapses under both lighting conditions. Together, these results suggest that ARR4 expression in cones requires STX3 and that phosphorylation of STX3 at T14 enhances its interaction with ARR4. Additionally, we could determine that in a heterozygous scenario of the cone‐specific STX3 depletion (*Stx3^f/+(HRGP‐Cre)^*), ARR4 remained detectable at cone OSs and synapses, with levels comparable to WT. Thus, 50% STX3 expression was sufficient to maintain integrity and ARR4 expression. The loss of ARR4 observed in homozygous *Stx3^f/f(HRGP‐Cre)^* cones may therefore reflect additional factors associated with cone degeneration in the absence of STX3.

The ribbon synapse features a specialized architecture that enables rapid, graded neurotransmission, distinct from conventional synapses that typically generate slower, all‐or‐none responses.^[^ ^14^, ^78^, ^79^ ^]^ This is particularly crucial for cone photoreceptors, which possess larger synaptic terminals and more ribbon synapses than rods, supporting their greater demand for dynamic vesicle release. In the *Nrl^−/−^* retina, loss of ARR4 enhanced photopic response, whereas ARR1 ablation reduced it.^[^ ^80^ ^]^ Combined deletion virtually attenuated cone responses, suggesting that ARR4 modulated or graded the synaptic response of cone photoreceptors, rather than generating the cone response itself. In vitro studies have shown that arrestins can transition between various cellular compartments upon activation,^[^ ^81^ ^]^ and it is possible that ARR4, while retained in cone terminals under both light and dark conditions, undergoes such conformational transitions, affecting its interaction with STX3. Since vesicle release is highest in darkness, when STX3 and ARR4 interact, this association may facilitate neurotransmitter release. This may occur by inducing a conformational switch in STX3 from a closed, inactive to an open, active state, or by promoting SNARE complex assembly to enable vesicle release.

**Figure 9:**
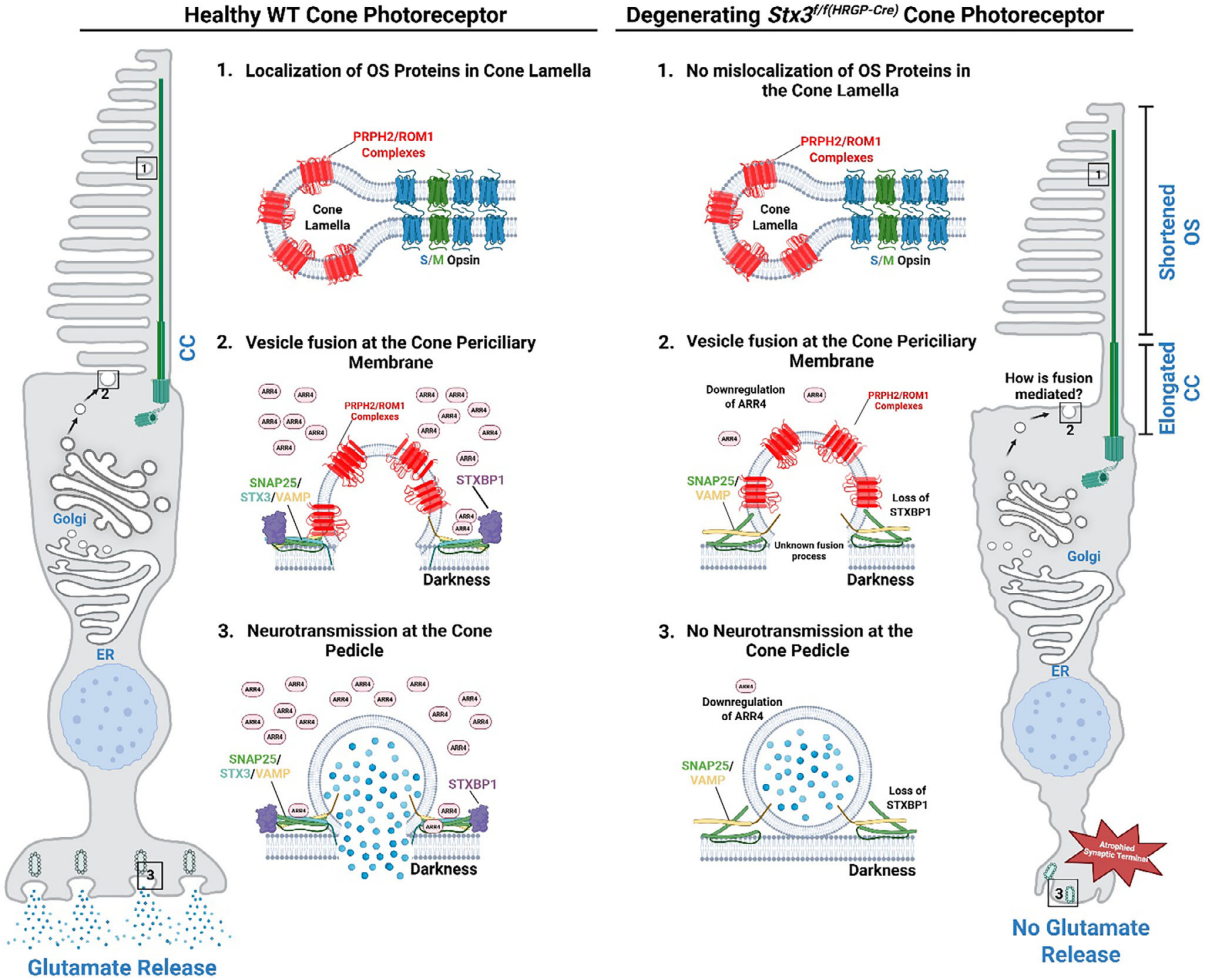
Graphical abstract summary. STX3 regulates cone photoreceptor synaptic function through interactions with STXBP1 and ARR4, both of which are lost upon cone‐specific STX3 depletion. While STX3 does not impact the transport of the essential OS proteins PRPH2, ROM1, and S‐ and M‐cone opsin, it is essential for synaptic integrity and vesicle release. Cone synapses, with their complex ribbon architecture, may require additional regulators like STXBP1 and ARR4 for precise release dynamics. These proteins show distinct regulation in rods versus cones, suggesting cell‐type‐specific mechanisms of STX3‐mediated exocytosis. Created with BioRender.com.

This study uncovers several cone‐specific characteristics of STX3, including the identification of two key interactors, STXBP1 and ARR4, the latter being newly described. Both proteins were depleted in the cones of the *Stx3^f/f(HRGP‐Cre)^* retina, in contrast to rods of the *Stx3^f/f(CRX‐Cre)^* model, where STXBP1 and ARR1 (the rod‐specific arrestin isoform) remained unaffected.^[^ ^27^ ^]^ Additional differences were observed in the localization of OS proteins: while PRPH2, ROM1, and cone opsins (S‐ and M‐opsin) remained properly localized in cones lacking STX3, their rod counterparts in *Stx3^f/f(CRX‐Cre)^* retina exhibited mislocalization. This suggests that STX3 is not essential for vesicle fusion at the ciliary base or OS protein trafficking in cones (**Figure** **9**). In contrast, synaptic function was profoundly impaired in cones lacking STX3. Cone pedicles displayed structural abnormalities, loss of STXBP1 and ARR4 (Figure 9). The complete absence of a photopic b‐wave indicates a lack of activity in depolarizing (ON) cone bipolar cells. Given the structural abnormalities and the depletion of STX3B, a key component of the SNARE complex responsible for vesicle release at photoreceptor ribbon synapses, at cone synapses, the absence of cone bipolar activity (postsynaptic to cone photoreceptors) is most likely caused by impaired synaptic vesicle release from cone synapses in the *Stx3^f/f(HRGP‐Cre)^* retina. Notably, ARR4 depletion in the *Nrl^−/−^* retina enhanced both photopic a and b‐wave amplitudes, suggesting a modulatory role in cone output.^[^ ^80^ ^]^ STXBP1, while present in both rod and cone synapses, also regulates vesicle release. However, whether it primarily facilitates SNARE complex assembly or inhibits exocytosis remains under debate. Importantly, STX3 loss in cones, but not in rods, results in the depletion of both ARR4 and STXBP1, implying that these proteins may play more critical or unique regulatory roles in cones. This dependency could reflect the more complex multi‐ribbon synaptic architecture of cones compared to a single ribbon in rods. Coordinated vesicle release across multiple ribbons likely demands additional regulatory mechanisms.^[^ ^52^, ^53^ ^]^ Our findings raise several outstanding questions. For instance, why is STXBP1 not co‐depleted in rods following STX3 loss, despite its strong localization to both rod and cone synapses in the *Stx3^f/f(CRX‐Cre)^* retina?^[^ ^27^ ^]^ Additionally, why is the STX3‐STXBP1 interaction disrupted in the dark‐adapted *Nrl^−/−^* retinas but preserved in WT rods? These observations point to distinct, context‐dependent regulatory mechanisms governing STX3‐mediated vesicle release in rods versus cones. Elucidating how STXBP1 and ARR4 influence STX3 function, and whether their actions are coordinated or independent, will be crucial for understanding the molecular logic underlying ribbon synapse function in photoreceptors.

## 4 Experimental Section

### Animals

All animal experiments in this study were approved by the University of Houston's Institutional Animal Care and Use Committee (IACUC) and adhered to the Association for Research in Vision and Ophthalmology (ARVO) guidelines. Animals were housed under cyclic light conditions (12 h light/dark cycles, at 30 lux) at a room temperature of 22 °C, with ad libitum access to food and water. For experiments involving light‐ and dark‐adaptation, animals were dark‐adapted overnight. For light exposure experiments, animals were anesthetized, pupils dilated, and placed on a heating pad (set to 37 °C) in a light box with a light intensity of 2500 lux for 2 h. Dark‐adapted animals were anesthetized, dilated in the dark, and placed on a heating pad for 2 h to ensure consistent treatment across experimental cohorts. Floxed STX3 mice (*Stx3^f/f^*) were generated by InGenious Targeting Laboratory, Inc. (Ronkonkoma, NY, USA) as previously described.^[^ ^27^ ^]^ For cone‐specific knockout of STX3, *Stx3^f/f^* mice were mated with *HRGP‐Cre* transgenic mice, developed and provided by Dr. Yun Le (University of Oklahoma Health Sciences Center).^[^ ^44^ ^]^ The *Stx3^f/f(CRX‐Cre)^* mice were described before.^[^ ^27^ ^]^ To investigate cone‐specific interactions between STX3 and other proteins, retinas were harvested from *Nrl^−/−^* mice.^[^ ^82^ ^]^ Retinal lysates from these mice were used for interaction assays such as immunoprecipitation and GST‐pulldown. Mice of both sexes were used in all experiments. Retina samples from *Arr1^−/−^* mice^[^ ^83^ ^]^ were kindly provided by Dr. Vladimir Kefalov (Gavin Herbert Eye Institute, University of California, Irvine, CA).

### Immunoblots

Retinas were collected, immediately frozen in liquid nitrogen, and stored at ‐80 °C before use. Two retinas were lysed in 100 µL 1xPBS (137 mm NaCl, 2.7 mm KCl, 1.5 mm KH_2_PO_4_, 8.1 mm Na_2_HPO_4_, pH 7.3) containing 1% Triton‐X‐100 (0694‐1L, VWR, Radnor, PA) and 1x protease inhibitor (Roche Applied Sciences, Indianapolis, IN). After the addition of lysis buffer, the retinas were sonicated in ten short bursts, incubated for 1 h on a rocking platform at 4 °C, and then spun at 18 000xg at 4 °C for 10 min. Protein concentration in the resulting supernatant was determined using a Bradford assay (Bio‐Rad Laboratories Inc., Hercules, CA). 4x Laemmli buffer (100 mm Tris‐Base pH 6.8, 4% SDS, 25% Glycerol, and 0.05% Bromophenol blue) was added to the samples (1 part 4x Laemmli buffer to 3 parts sample), followed by 5 min boiling at 95 °C. After a brief spin, the samples were loaded onto SDS‐PAGE gels.

SDS‐PAGE gels were cast with polyacrylamide concentrations ranging from 11 to 15% and run in 1x running buffer (25 mm Tris‐base, 200 mm Glycine, 0.1% SDS, pH 8.3). Proteins were transferred using a Trans‐Blot Turbo (Bio‐Rad Laboratories Inc.) semidry blot system with 1x transfer buffer (25 mm Tris base, 200 mm Glycine, 0.1% SDS, 10% MeOH). Samples analyzed following GST‐Pulldown (see below) were transferred to a PVDF membrane (IEVH00005, Sigma Aldrich, St. Louis, MO) using a Mini Trans‐Blot Cell (Bio‐Rad Laboratories Inc.) at 100 V for 90 min in 1x wet transfer buffer (25 mm Tris base, 200 mm Glycine, 0.01% SDS, 20% MeOH). After transfer, the membrane was blocked in 5% non‐fat dry milk dissolved in 1xPBS with 0.1% Tween20 (P7949, Sigma Aldrich). Primary antibodies (diluted in blocking solution, as detailed in Supplementary Table S1) were applied overnight at 4 °C. Secondary antibodies against mouse, rabbit, and goat, conjugated to HRP, diluted 1:15 000 in blocking solution, were applied for 2 h at room temperature. Protein detection was carried out using SuperSignal West Pico plus chemiluminescent substrate (34580, Thermo Fisher Scientific, Waltham, MA) and visualized on a ChemiDoc MP Imager (Image Lab, Version 3.0.1.14, Bio‐Rad Laboratories Inc.) and quantified using Image Lab 6.1 software (Version 6.1.0 build 7, BioRad Laboratories Inc).

### Immunoprecipitation

For the IPs in Figure 8A, flash‐frozen retinas from light‐ and dark‐adapted WT animals were sonicated (10‐12 short pulses) in HEPES buffer containing CHAPS (20 mm HEPES pH 7.4, 150 mm NaCl, 10 mm CHAPS, and protease inhibitor). Following sonication, the samples were incubated on a nutator at 4 °C overnight to ensure effective solubilization. The next day, the samples were centrifuged at 12 000xg for 15 min, and the protein concentration of the supernatant was determined by a Bradford Assay (Bio‐Rad Laboratories Inc.). 200 µg of protein were used for immunoprecipitation, as previously described.^[^ ^84^ ^]^ To pull down the antigen‐anti‐Syntaxin 3B complex, protein G beads (Thermo Fisher Scientific) were used. “Input” (20% of total protein) and one‐third of the final elution were loaded onto 12% polyacrylamide gels for subsequent analysis by immunoblotting. For Figure 8E, lysis was conducted in 1xPBS pH 7.3 containing 10 mm CHAPS. Lysis after sonication was done for 1 h at 4 °C. 10% of the total protein was used as input, while half of the final elution was loaded onto 12% polyacrylamide gels (for STXBP1 probing) and onto 15% polyacrylamide gels (STX3B and SNAP25 probing).

### GST‐Pulldown

Retinas from light‐ and dark‐adapted WT and *Nrl^−/−^* mice were collected, flash frozen in liquid nitrogen, and stored at ‐80 °C until use. Retina extraction was performed by homogenizing two frozen retinas in 100 µL lysis buffer (10 mm CHAPS, 5 mm EDTA in 1xPBS pH 7.3) containing 1x protease inhibitors (Roche Applied Sciences). The samples were sonicated with 10 short pulses and incubated for 1 h on a rocking platform at 4 °C. To remove insoluble debris, the lysate was centrifuged at 18,000xg at 4 °C for 10 min. Protein concentration was determined using the Bradford assay.

Recombinant Glutathione‐S‐transferase (GST, expressed from pGEX4T2) and GST‐tagged STX3 lacking the transmembrane domain (GST‐STX3‐Δtm, generously provided by Dr. Roger Janz, McGovern Medical School, UTHealth, Houston, TX) were expressed in *E.coli* strain BL21. LB media was inoculated with BL21 bacteria transformed with the GST constructs described above, incubated at 37 °C till OD_600_ = 0.6 to 0.8, induced with 0.5 mM IPTG (15529019, Thermo Fisher Scientific, Waltham, MA), and incubated at 37 °C after induction. After induction, the bacteria were incubated at 37 °C for 3 h, pelleted at 3,220xg, and stored overnight at ‐80 °C. The following day, the bacteria pellet was thawed 10 min at room temperature and resuspended in 22.5 mL 1xPBS pH 7.3 with 1x protease inhibitor (Roche Applied Sciences). All subsequent steps were performed on ice. After the addition of 2.5 mL of lysozyme solution (10 mg mL^−1^ lysozyme from chicken egg; L6876, Sigma Aldrich, St. Louis, MO) solved in 25 mm Tris‐base pH 8.0), the sample was then incubated for 15 min on ice. The sample was then treated with 10% sarkosyl (L‐9150, Sigma Aldrich) dissolved in 1xSTE buffer (10 mm Tris‐HCL, pH 8.0, 1 mm EDTA, 150 mm NaCl), sonicated (two 30 s bursts with 30 s on ice in between), and incubated on ice for another 15 min. After the addition of 2.5 mL of 10% Triton‐X‐100 (VWR) diluted in 1xSTE buffer, the sample was incubated for 1 h in an overhead shaker at 4 °C. The lysate was then centrifuged at 16 000xg for 1 h at 4 °C, and the resulting supernatant was used in the GST‐pulldown.

Glutathione Sepharose (GS) beads (50 µL per sample; 17‐0756‐01, GE HealthCare, Chicago, IL) were washed three times with 500 µL NETN buffer (20 mm Tris, pH 8.0, 100 mm NaCl, 1 mm EDTA, 0.5% NP‐40) per sample. To bind GST proteins, washed GS beads were incubated with GST‐proteins diluted in NETN buffer, achieving a total volume of 100 µL, for 2 h at 4 °C on an overhead shaker. Preclearing of the retina lysates was achieved by incubating the retina lysates for 1 h with GS beads bound with GST. GS beads with the bound GST constructs were then washed three times in 500 µL lysis buffer (see above). The precleared lysate was added to the washed GS beads and incubated overnight at 4 °C on an overhead shaker. The following day, beads were washed three times in 500 µL lysis buffer. Bound proteins were eluted by adding 80 µL of 4x Laemmli buffer containing 10% β‐mercaptoethanol, followed by 20 min of incubation at room temperature on an overhead shaker. Eluted proteins were then boiled for 10 min at 95 °C and centrifuged twice at 18000xg for 10 min each at 40 °C. The eluted proteins and 1% of the input were loaded onto SDS‐PAGE gels for subsequent analysis via immunoblotting as described above.

### Immunofluorescence

Mouse eyes were marked on the superior hemisphere using a cauterizer (Aspen Surgical, Inc., Caledonia, MI), enucleated post euthanasia, and fixed in modified Davidson fixative (9% PFA, 33% EtOH, 11% acetic acid) for 6 h or 4% PFA for 3 h. Fixed eyes were then paraffin‐embedded (STP 120 Spin Tissue Processor, Thermo Fisher Scientific). Embedded eyes were sectioned at 5 µm for general immunofluorescence and 10 µm for cone cell counts using a HM 355 S Automatic Microtome (Thermo Fisher Scientific). Sections were collected on microscope slides and stored at room temperature until use. For immunolabelling, sections were deparaffinized for 30 min in xylene, followed by rehydration in a graded ethanol series (100, 90, 80, 70, and 50%, 5 min for each step). After two 5 min washes in water, antigen retrieval was performed by boiling sections in Tris/EDTA buffer (10 mm Tris Base pH 9.0, 1 mm EDTA Solution, 0.05% Tween 20) for 30 min, followed by a 10 min cool‐down at room temperature. Sections were then washed twice in water (3 min each), quenched for autofluorescence in 1% NaBH_4_ for 3 min, and washed twice in water (3 min each). Following two 5 min washes in 1xPBS, sections were blocked for 30–60 min in blocking solution (0.5% Triton X‐100, 2.5% donkey serum, 5% BSA, and 1% fish gelatin (when labeling with anti‐ARR1 and anti‐ARR4) in dissolved 1x PBS). Primary antibodies were applied overnight at 4 °C in blocking solution. The next day, the sections were washed three times in 1xPBS for 10 min each and incubated with secondary antibodies diluted 1:500 in blocking solution for 2 h at room temperature. Sections then underwent three additional 10 min washes in 1xPBS, followed by a 30 min incubation with DAPI (Thermo Fisher Scientific, 1:500 in blocking solution). After three additional 10 min washes in 1xPBS, the sections were mounted using Prolong Gold antifade mounting reagent (P36934, Thermo Fisher Scientific). Images were acquired using a Zeiss LSM 800 (Zen 2.3, version 2.3.69.1018, Carl Zeiss Microscopy GmbH, Jena, Germany) and further processed using Zen lite 3.9 (version 3.9.101.04000, Zeiss Microscopy GmbH). The following secondary antibodies were used: Alexa Fluor‐488 donkey‐anti‐mouse (A21206), Alexa Fluor‐555 goat‐anti‐rabbit (A21428), and Alexa Fluor‐647 donkey‐anti‐rabbit (A31573), all purchased from Thermo Fisher Scientific. In certain experiments, PNA conjugated to Cy3 (CL‐1073, Vector Laboratories Inc., Newark, CA) was added at a 1:40 dilution to label cone glycocalyx.

### Dissociation of Cone Outer and Inner Segments

Fresh retinas were collected from WT and *Stx3^f/f(HRGP‐Cre^* ^)^ mice and placed in 125 µL of cold 1X PBS. Samples were vortexed at maximum speed (Vortex Genie 2, Scientific Industries, Pasadena, TX) for 6‐7 pulses of 10 s each. Retinas were then centrifuged at 100×g for 1 min, and the supernatant was transferred onto charged slides within circular regions demarcated by a PAP pen. The retinal pellets were resuspended in 125 µL of 1X PBS and vortexed again at maximum speed for 1 min. The mixture was centrifuged at 100×g for 1 min, and the resulting supernatant was again applied to slides as described above. Slides were left undisturbed for 20 min, after which excess liquid was removed with a pipette and discarded. Samples were fixed in cold methanol for 10 min, followed by two washes in water and two washes in 1X PBS. Blocking buffer was then applied, and standard immunofluorescence was performed as described above. Cones retaining both inner and outer segments were observed primarily on slides prepared from the second 1 min vortex.

### Cone Cell Counts and Measurement of Cone Cilia Length

Retinal sections (10 µm thickness) were prepared as described above, except antigen retrieval was done for 8 min instead of 30. M‐ and S‐cones were labeled with M‐ and S‐opsin antibodies. Entire sections were imaged using Zeiss LSM 800 (Zen 2.3, version 2.3.69.1018, Carl Zeiss Microscopy GmbH) by tiling images taken with a 20x objective. For cone quantifications, S‐ and M‐cones were counted in the central inferior and superior regions, spanning 350 µm of the section, starting 300 µm from the optic nerve center. Total cone counts were determined by counting M‐ and S‐cones across the entire section. Data were collected from three to four tiled images per section, with three to four mice per genotype and age group. Measuring the distances and counting the cones was done using ImageJ (version 1.53f51, NIH).

Eyes from P30 and P90 WT and *Stx3^f/f(HRGP‐Cre)^* mice were collected and fixed in 4% PFA for 15 min, a small incision was made, and the eyes were returned to the fixative. After 1 h of fixation, the retina was dissected and further fixed for 1 h. Retinas were then washed 3x in 1XPBS for 10 min each, blocked for 1 h in blocking solution (see above in immunofluorescence), and then incubated overnight at 4 °C with anti‐S‐opsin antibody. The following day, retinas were washed 3 times in 1XPBS (10 min each) and incubated for 2 h with Alexa Fluor‐488 donkey‐anti‐rabbit antibody (A21206, Thermo Fisher Scientific, 1:500 dilution), PNA conjugated to Cy3 (CL‐1073, Vector Laboratories, 1:200 dilution), and DAPI (Thermo Fisher Scientific, 1:8000 dilution). Retinas were then washed 3 times with 1XPBS (10 min each), butterflied, and mounted with Prolong Gold antifade mounting reagent (P36934, Thermo Fisher Scientific). Whole mounts were imaged using a Zeiss LSM 800 confocal microscope (Zen 2.3, version 2.3.69.1018, Carl Zeiss Microscopy GmbH) with tiled images acquired using a 20x objective.

For cone cilia measurements, 10 µm sections were labeled for S‐ and M‐opsin to identify respective cone types, while acetylated tubulin was used to label cilia. Cilia were measured in images obtained with a Zeiss LSM 800 (Zen 2.3, version 2.3.69.1018, Carl Zeiss Microscopy GmbH) using a 63x objective. Cilia length was measured using ImageJ in images obtained from three P30 mice per genotype.

### Electroretinography

Mice were dark‐adapted overnight and anesthetized using 85 mg kg^−1^ ketamine and 14 mg kg^−1^ xylazine (Butler Schein Animal Health, Dublin, OH, USA).^[^ ^85^, ^86^, ^87^, ^88^ ^]^ Dilation of the eyes was achieved using a 1% cyclogyl solution (Akorn, Lake Forest, IL, USA). Full‐field ERG was assessed with the UTAS system (LKC, Gaithersburg, MD, USA; software: EMwin, Version 9.8.0). The platinum wire electrode was in contact with the cornea through a layer of Gonak (Akorn). A single strobe flash stimulus of 157 cd‐s m^−2^ was presented to dark‐adapted mice to measure the scotopic response, followed by 5 min light exposure via background light with an intensity of 30 cd m^−2^. The photopic response was measured by averaging the responses of 25 flashes of white light at 90 cd‐s m^−2^.

### Histology

Eyes were collected, fixed, and paraffin‐embedded as described in the Immunofluorescence section. Retinal sections (10 µm thickness) were prepared using an HM 355 S Automatic Microtome (Thermo Fisher Scientific). For rehydration, sections were incubated twice in xylene (30 min each), followed by 100% EtOH for 10 min. A graded ethanol series (90, 80, 70, and 50%, 5 min per step) was then applied, followed by a 10 min wash in water. Nuclei were stained with hematoxylin (MHS16, Sigma Aldrich) and eosin (HT110116, Sigma Aldrich). Sections were mounted using Permount mounting medium (SP15100, Thermo Fisher Scientific) and imaged using the 40x objective on a Zeiss Axioskop 50 (Zen 2.0, Version 2.0.0.0, Carl Zeiss, White Plains, NY). Images were taken 500 µm from the optic nerve. ONL nuclei were quantified in a 100 µm window at 200 µm intervals along the superior‐inferior axis using Zen lite 3.9 (Version 3.9.101.04000, Zeiss Microscopy GmbH) and ImageJ (version 1.53f51, NIH).

### Study Approval

All handling, maintenance, and experimental use of animals followed protocols (Protocol: PROTO20200007) approved by the University of Houston's Institutional Animal Care and Use Committees and were performed according to the NIH and the Association for Research in Vision and Ophthalmology (ARVO) guidelines.

### Statistical Analyses

Quantification of STX3B levels in Figure 1D was analyzed using a two‐tailed unpaired t‐test. For the ERG measurement, spider graphs for ONL nuclei count and cone counts, significance was determined using a two‐way ANOVA with Sidak's post hoc comparison. Cone cilia on OS length measurements were analyzed using a two‐tailed unpaired *t*‐test. Quantifiable immunoblots in Figures 6 and 7 were analyzed using a two‐tailed unpaired *t*‐test with Welch's correction. All statistical analyses and graph plotting were performed using Prism 7 (software version 7.05, GraphPad Software Inc., Boston, MA).

### Additional Software

All figures except Figures 3E and 9 were assembled using Photoshop 2024 (Adobe, San Jose, CA, USA; version 25.9.0). Figures 3E and 9 were assembled using the web tool BioRender (biorender.com).

## Conflict of Interest

The authors declare no conflict of interest.

## Author Contributions

L. T., L. I. contributed equally to this work. LT, LI, MRA, and MIN conceived and designed the experiments. LT and LI performed the experiments, analyzed the data, and prepared the illustrations for the manuscript. LT wrote the initial draft. MK performed the immunofluorescence presented in Figure 5. MSM carried out the ERG measurements and morphometric analyses of *Stx3^f/f(HRGP‐Cre)^* mice, shown in Figure 2 and Figure S1. MRA and MIN revised and finalized the manuscript.

## Supporting information



Supporting Information

Supporting Information

## Data Availability

The data that support the findings of this study are available in the supplementary material of this article.
