# Supplementary material for: Syntaxin 3B Mediates Light‐Dependent Interactions with STXBP1 and Arrestin 4: Distinct Roles in Rods and Cones
Source: Adv Sci (Weinh). 2025 Nov 12;13(6):e13319. doi: 10.1002/advs.202513319 (PMC12866690; doi:10.1002/advs.202513319)
Supplement: Supplementary file 1 — Supporting Information [file ADVS-13-e13319-s001.pdf]

Syntaxin 3B Mediates Light-Dependent Interactions with STXBP1 and Arrestin 4: Distinct Roles in Rods and Cones

Lars Tebbe[1], Larissa Ikelle[1], Mustafa S. Makia, Mashal Kakakhel, Muayyad R. Al-Ubaidi[*] and Muna I. Naash[*]

Department of Biomedical Engineering, University of Houston, Houston, TX 77204, USA.

[1]Equal contributors

[*]To whom correspondence should be addressed at: Muna I. Naash (ORCID: 0000-0002-6534-5144), mnaash@central.uh.edu; Phone: 713-743-1651 or Muayyad R. Al-Ubaidi, (ORCID 0000-0002-4914-350X), malubaid@central.uh.edu; Phone: 713-743-1648; Department of Biomedical Engineering, University of Houston, 3517 Cullen Blvd. Room 2027, Houston, TX 77204-5060.

Supplementary material

Supplementary Table 1. Primary antibodies used in the study. Abbreviation: IF: Immunofluorescence; IB: immunoblot.

Antigen	Species	Application/concentration	Source	RRID#
Acetylated tubulin	mouse	1:400 (IF)	Sigma Aldrich, (T7451)	AB_609894
Actin (-HRP)	mouse	1:15000 (IB)	Sigma Aldrich, (A3854)	AB_262011
ARR1	mouse	1:100 (IF), 1:1000 (IB)	MH785, gift from Dr. Paul Hargrave[1]	NA
ARR4	rabbit	1:100 (IF), 1:1000 (IB)	Gift from Dr. Cheryl Craft[2]	NA
ARR4	rabbit	1:250 (IF), 1:1000 (IB)	Millipore Sigma, (AB15282)	AB_1163387
FLAG M2	mouse	1:1000 (IB)	Sigma Aldrich, (F1804)	AB_262044
GST	goat	1:1000 (IB)	GE Healthcare, (27-4577-01)	AB_771432
M-Opsin	rabbit	1:400 (IF)	Sigma Aldrich, (AB5405)	AB_177456
PRPH2	rabbit	1:400 (IF), 1:1000 (IB)	In House[3]	AB_2833006
PRPH2	mouse	1:400 (IF), 1:1000 (IB)	2B7, in house[4]	NA
ROM1	mouse	1:100 (IF)	2H5, in house[4]	NA
SNAP25	rabbit	1:1000 (IB)	Proteintech, (14903-1-AP)	AB_2192051
S-Opsin	rabbit	1:400 (IF)	Sigma Aldrich, (AB5407)	AB_177457
STX3	rabbit	1:100 (IF)	Abcam, (AB4113)	AB_2198665
STX3B	mouse	1:100 (IF), 1:1000 (IB)	12E5, in House[5]	NA
STXBP1	rabbit	1:100 (IF)	Proteintech, (20562-1-AP)	AB_10694824
STXBP1	mouse	1:100 (IF), 1:1000 (IB)	Proteintech, (67137-1-Ig)	AB_2882436

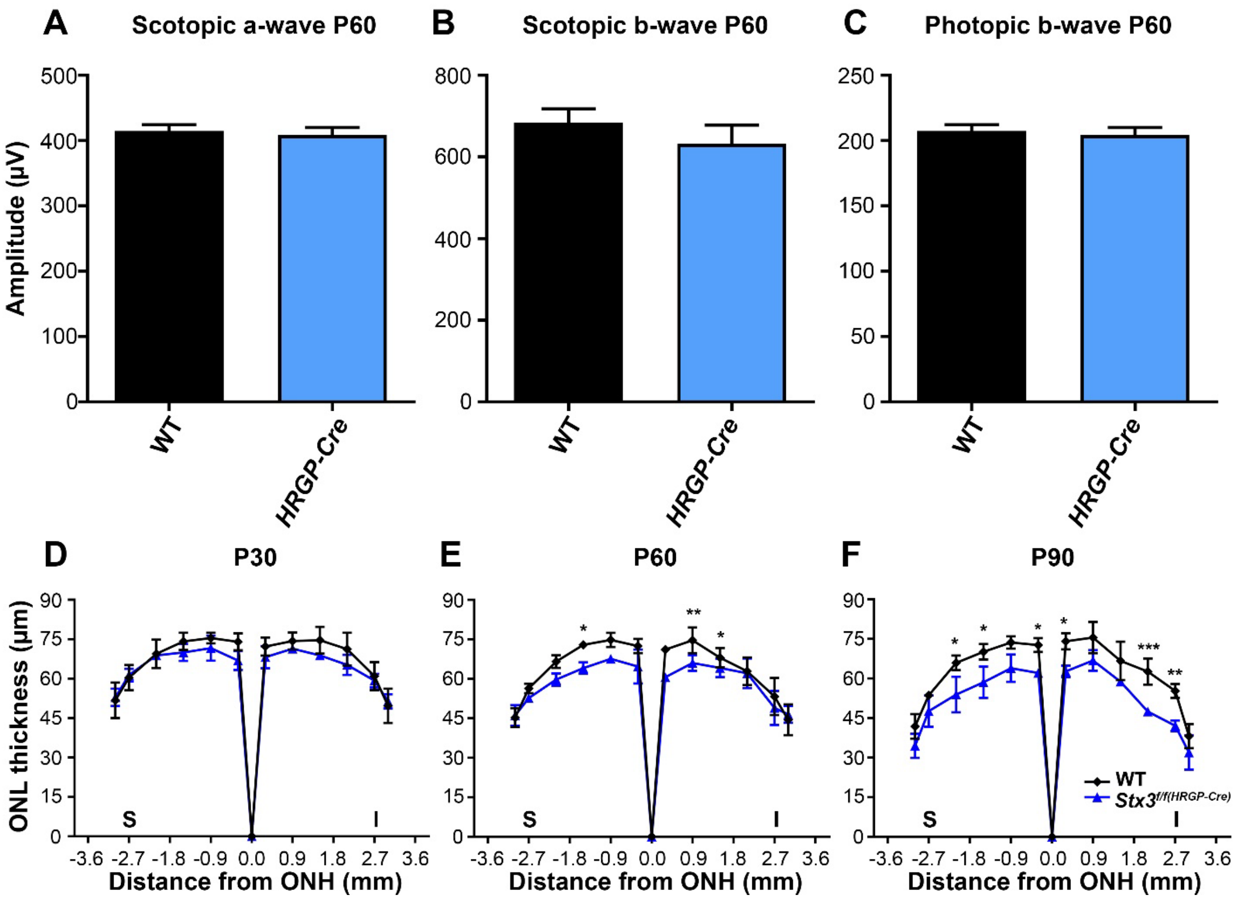

Supplementary Figure 1. Expression of HRGP-Cre does not impact retinal function but depletion of STX3 in cones impacts ONL thickness. Maximum amplitudes of scotopic a-waves (**A**), scotopic b-waves (**B**), and photopic b-waves (**C**) in *HRGP-Cre* mice at P60. No significant differences were observed. N-values: Scotopic a-wave: WT: 6 mice; *HRGP-Cre*: 5 mice. Scotopic b-wave: WT: 6 mice; *HRGP-Cre*: 5 mice. Photopic b-wave: WT: 6 mice; *HRGP-Cre*: 5 mice Data are presented as mean ± SEM. P-values determined by two-tailed unpaired t-test. **(D-F)** Quantification of ONL thickness at P30 (D), P60 (E), and P90 (F) shows a significant reduction in ONL thickness starting at P60 in the $Stx3^{f/f(HRGP\text{-}Cre)}$ retina, with further progression by P90. N-values: WT: 3 mice for P30 and P90, 4 mice for P60. $Stx3^{f/f(HRGP\text{-}Cre)}$: 3 mice for P30, P60, and P90. Data are presented as mean ± SEM. Significant P-values: P60: -1.5 mm: 0.037; 0,3 mm: 0.0062; 0.9 mm: 0.0494. P90: -2.1 mm: 0.0101; -1.5 mm: 0.0159; -0.3 mm: 0.0345; 0.3 mm: 0.0184; 2.1 mm: 0.0005; 2.7 mm: 0.0034. Abbreviations: ONL: outer nuclear layer; ONH: optic nerve head; I: inferior; S: superior.

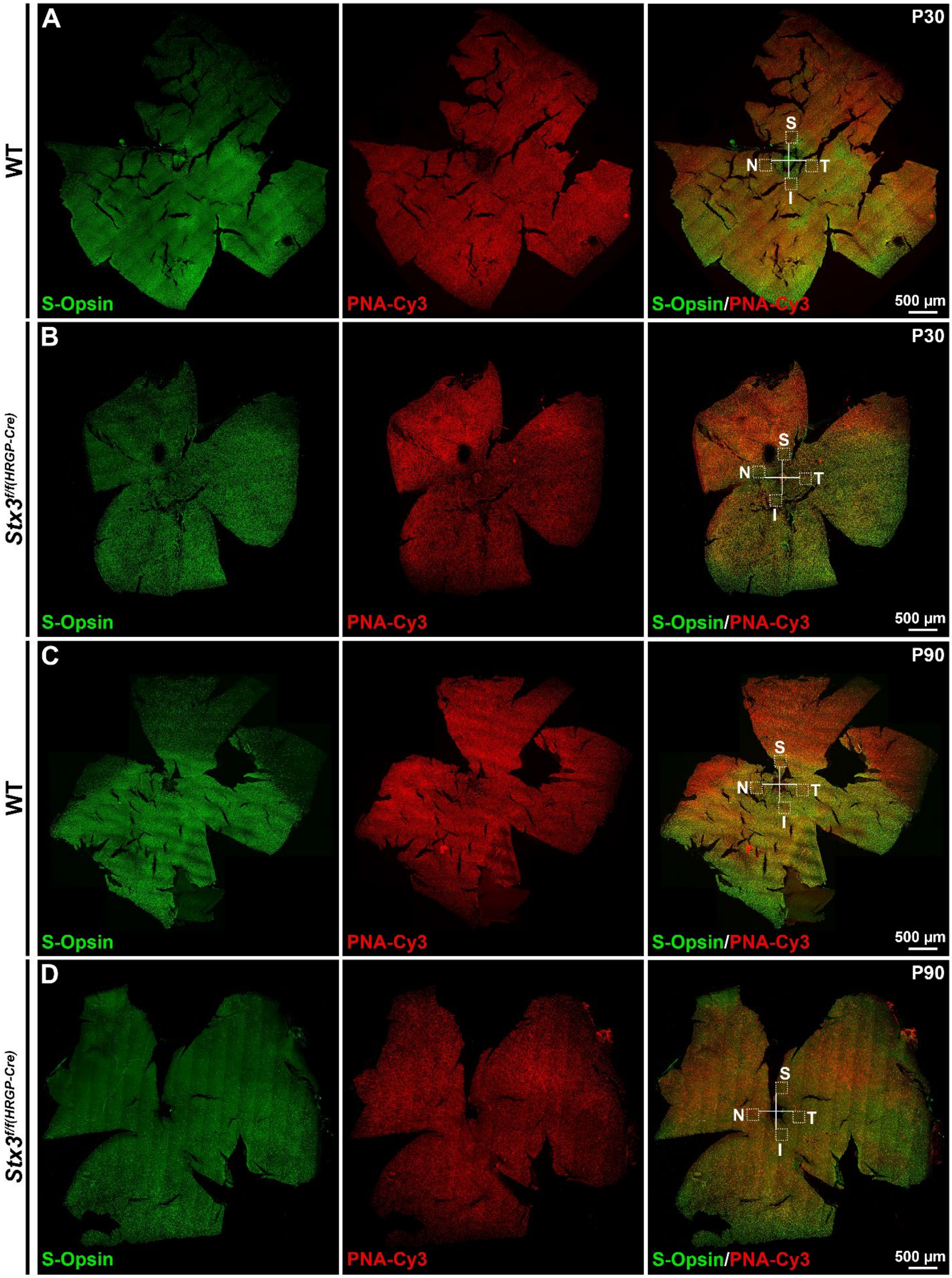
A
P30
WT
S-Opsin
PNA-Cy3
S-Opsin/PNA-Cy3
500 μm
S
N
T
I
B
P30
Stx3f/f(HRGP-Cre)
S-Opsin
PNA-Cy3
S-Opsin/PNA-Cy3
500 μm
C
P90
WT
S-Opsin
PNA-Cy3
S-Opsin/PNA-Cy3
500 μm
D
P90
Stx3f/f(HRGP-Cre)
S-Opsin
PNA-Cy3
S-Opsin/PNA-Cy3
500 μm

Supplementary Figure 2. Wholemounts demonstrating progressive cone loss in the *Stx3*$^{f/f(HRGP\text{-}Cre)}$ retinas. A-D) Overview of the complete wholemounts used in Figure 3 F and G. WT (A) and *Stx3*$^{f/f(HRGP\text{-}Cre)}$ (B) retinas labelled for S-Opsin (S-cones) and PNA-Cy3 (total cones) at P30 show loss of S-cones and total cones in *Stx3*$^{f/f(HRGP\text{-}Cre)}$ retinas. **C and D**) Wholemounts of WT (C) and *Stx3*$^{f/f(HRGP\text{-}Cre)}$ (D) retinas at P90, reveal further progression of cone loss in *Stx3*$^{f/f(HRGP\text{-}Cre)}$ retinas. Areas highlighted with boxes indicate the regions of the insets shown in Figure3 F and G. Abbreviations: S: superior; T: temporal; I: inferior; N: nasal.

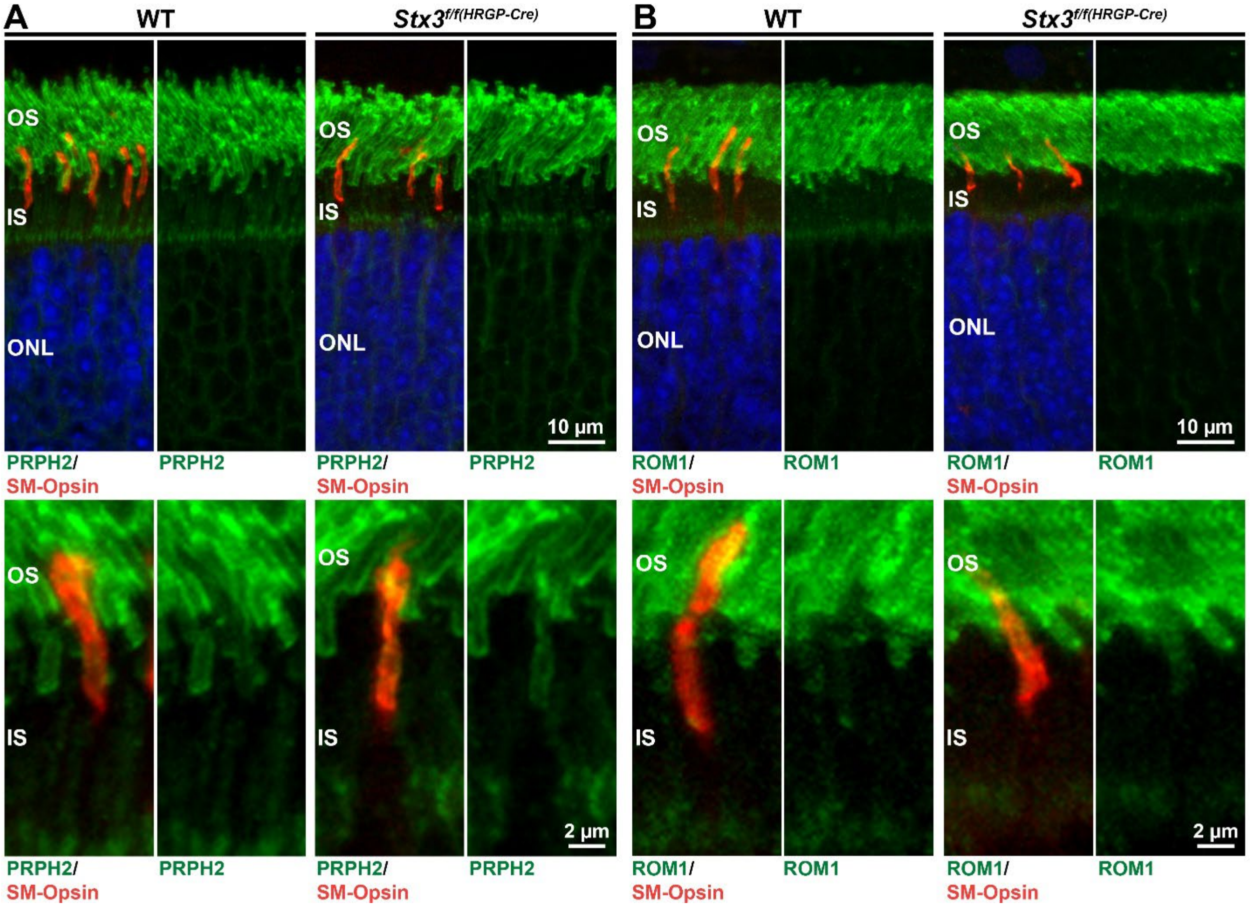

Supplementary Figure 3. Essential outer segment proteins are not mislocalized in the cones of the *Stx3$^{f/f(HRGP\text{-}Cre)}$* retina. A) Co-labeling for S- and M-Opsins (SM-Opsin) and for PRPH2 in P30 WT (left panels) and *Stx3$^{f/f(HRGP\text{-}Cre)}$* (right panels) mouse retinas. No mislocalization of PRPH2 or cone opsins is observed. **B)** WT (left) and *Stx3$^{f/f(HRGP\text{-}Cre)}$* (right) P30 mice, co-labeled for SM-Opsin and ROM1. No mislocalization is observed. Abbreviations: OS: outer segment; IS: inner segment; ONL: outer nuclear layer.

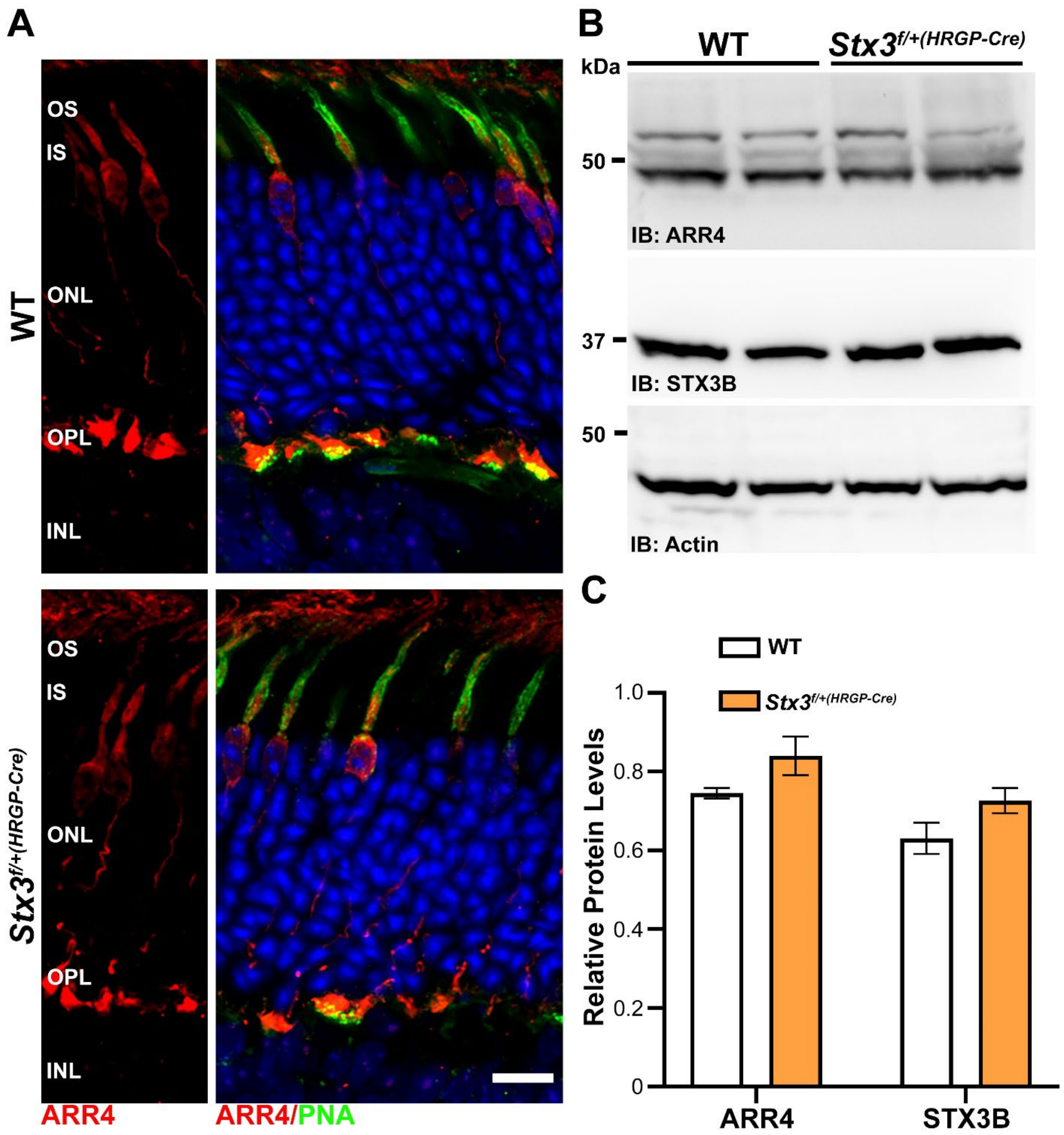

Supplementary Figure 4. **ARR4 expression and localization are maintained in *Stx3*$^{f/+(HRGP-Cre)}$ Mice. A)** Retinas of WT and *Stx3*$^{f/+(HRGP-Cre)}$ animals were collected under cyclic light and were labeled for ARR4 (red) and PNA (green). In both cohorts, ARR4 is correctly localized at the cone pedicle, throughout the ONL and in cone IS and OSs. Images were acquired at 63X magnification and are presented as collapsed confocal stacks. **B)** Retinas were also immunoblotted to assess changes to ARR4 and STX3B. **C)** Quantification of immunoblots from B indicates no change in

protein expression levels between cohorts. N=2. Abbreviations: OS: outer segment; IS: inner segment; ONL: outer nuclear layer; OPL: outer plexiform layer; INL: inner nuclear layer.

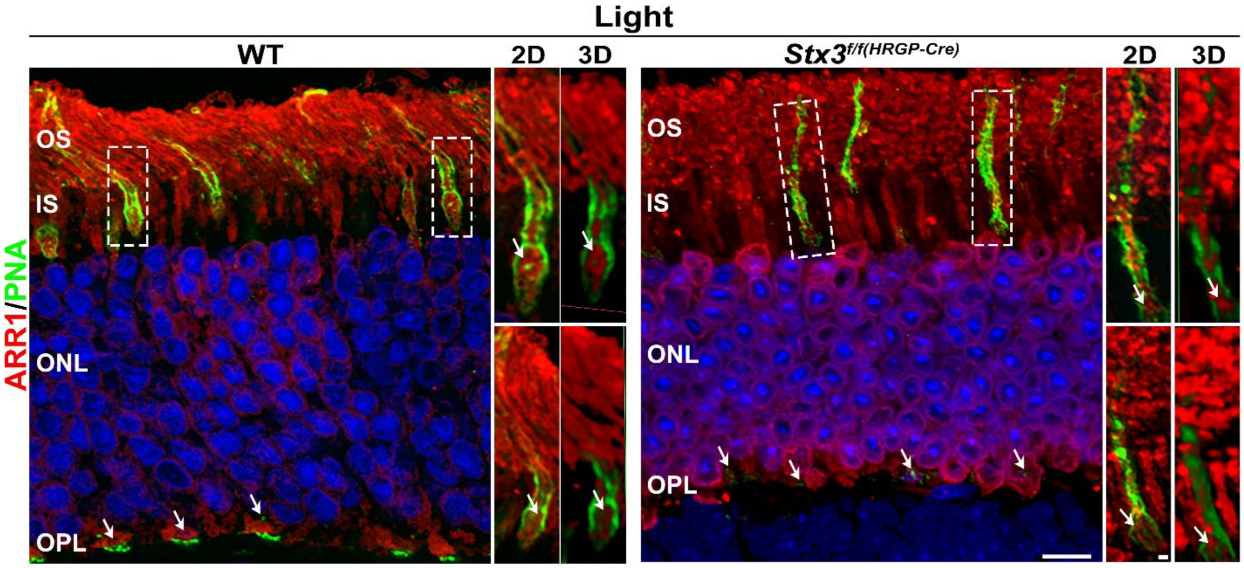

Supplementary Figure 5. **ARR1 localization is unchanged in the** ***Stx3$^{f/f(HRGP\text{-}Cre)}$*** **model.** Retinal cross-sections from light-adapted P30 WT and *Stx3$^{f/f(HRGP\text{-}Cre)}$* mouse retinas were labeled for ARR1 (red) and PNA (green). ARR1 in cone inner segments and cone pedicles are highlighted with arrows. Images were acquired at 63X magnification and are presented as collapsed confocal stacks. Cone photoreceptors, highlighted by white boxes, are shown in both 2D and 3D renderings (right panels). Scale bars: 10 µm in large panels, 0.5 µm in 2D and 3D panels. OS: outer segment, IS: inner segment, ONL: outer nuclear layer, and OPL: outer plexiform layer.

1. Elias, R.V., et al., *Temporal kinetics of the light/dark translocation and compartmentation of arrestin and alpha-transducin in mouse photoreceptor cells.* Mol Vis, 2004. **10**: p. 672-81.
2. Zhu, X., et al., *Mouse cone arrestin expression pattern: light induced translocation in cone photoreceptors.* Mol Vis, 2002. **8**: p. 462-71.
3. Ding, X.Q., H.M. Stricker, and M.I. Naash, *Role of the second intradiscal loop of peripherin/rds in homo and hetero associations.* Biochemistry, 2005. **44**(12): p. 4897-904.
4. Conley, S.M., et al., *Insights into the mechanisms of macular degeneration associated with the R172W mutation in RDS.* Hum Mol Genet, 2014. **23**(12): p. 3102-14.
5. Zulliger, R., et al., *SNAREs Interact with Retinal Degeneration Slow and Rod Outer Segment Membrane Protein-1 during Conventional and Unconventional Outer Segment Targeting.* PLoS One, 2015. **10**(9): p. e0138508.
